# L1 Cell Adhesion Molecule Confers Radioresistance to Ovarian Cancer and Defines a New Cancer Stem Cell Population

**DOI:** 10.3390/cancers12010217

**Published:** 2020-01-15

**Authors:** Nastassja Terraneo, Francis Jacob, Claudia Peitzsch, Anna Dubrovska, Christiane Krudewig, Yen-Lin Huang, Viola Heinzelmann-Schwarz, Roger Schibli, Martin Béhé, Jürgen Grünberg

**Affiliations:** 1 Center for Radiopharmaceutical Sciences ETH-PSI-USZ, Paul Scherrer Institute, 5232 Villigen-PSI, Switzerland; nastassja.terraneo@psi.ch (N.T.); roger.schibli@psi.ch (R.S.); martin.behe@psi.ch (M.B.); 2 Ovarian Cancer Research, Department of Biomedicine, University Hospital Basel and University of Basel, 4031 Basel, Switzerland; francis.jacob@unibas.ch (F.J.); y.huang@unibas.ch (Y.-L.H.); viola.heinzelmann@usb.ch (V.H.-S.); 3 National Center for Tumor Diseases (NCT), Partner Site Dresden, 01307 Dresden, Germany; claudia.peitzsch@nct-dresden.de; 4 German Cancer Research Center (DKFZ), 69120 Heidelberg, Germany; 5 OncoRay-National Center for Radiation Research in Oncology, Faculty of Medicine and University Hospital Carl Gustav Carus, Technische Universität Dresden, 01307 Dresden, Germany; anna.dubrovska@oncoray.de; 6 German Cancer Consortium (DKTK), Partner site Dresden, 01307 Dresden, Germany; 7 Helmholtz-Zentrum Dresden-Rossendorf, Institute of Radiooncology-OncoRay, 01307 Dresden, Germany; 8 Laboratory for Animal Model Pathology (LAMP), Institute of Veterinary Pathology, Vetsuisse Faculty, University of Zurich, 8057 Zurich, Switzerland; christiane.krudewig@uzh.ch; 9 Hospital for Women, University Hospital Basel, 4031 Basel, Switzerland; 10 Department of Chemistry and Applied Biosciences, ETH Zurich, 8093 Zurich, Switzerland

**Keywords:** L1 cell adhesion molecule, ovarian cancer, stem cells, radioresistance, CRISPR-Cas9, epithelial-to-mesenchymal transition

## Abstract

Many solid tumors, including ovarian cancer, contain small populations of cancer stem cells (CSCs). These cells are usually resistant against conventional cancer therapies and play a role in disease recurrence. We demonstrated that the L1 cell adhesion molecule (L1CAM) is a new CSC target in ovarian cancer, triggering radioresistance. Using fluorescence-activated cell sorting, specific cell populations expressing L1CAM alone or in combination with the established CSC marker CD133 were isolated from three ovarian cancer cell lines. Double-positive L1CAM+/CD133+ cells displayed higher spherogenic and clonogenic properties in comparison to L1CAM−/CD133− cells. Furthermore, L1CAM+/CD133+ cells retained highest clonogenic capacity after irradiation and exhibited up-regulation of some CSC-specific genes, enhanced tumor-initiating capacity, self-renewal and higher tumor take rate in nude mice when compared with other cell populations. Superior radioresistance by L1CAM expression was confirmed by deletion of L1CAM using CRISPR-Cas9 technology. Moreover, we found expression signatures associated with epithelial-to-mesenchymal transition phenotype in L1CAM deleted cells. These results indicate that L1CAM in combination with CD133 defines a new cancer cell population of ovarian tumor-initiating cells with the implication of targeting L1CAM as a novel therapeutic approach for ovarian CSCs.

## 1. Introduction

Ovarian cancer (OC) is the fifth most common type of cancer in females and one of the most lethal gynecologic tumors [1,2]. Because of the absence of specific symptoms and the lack of efficient screening methods, the majority of patients are diagnosed only at the advanced stage of the disease (FIGO stage III or stage IV), when metastases have already spread in the adjacent organs and in the peritoneal cavity [3]. Standard treatment for advanced OC includes cytoreductive surgery combined with platinum- and taxane-based chemotherapy. Despite a good response rate towards first-line chemotherapy (50–80%), most patients relapse within 5 years, developing therapy-resistant tumors with a median progression-free survival of 18 months [4,5]. In recent years, targeted therapies including both antibodies and specific tyrosine kinase inhibitors have been employed to treat OC; however, the clinical benefits have been limited so far [6]. There is persuading evidence that OC contains distinct subpopulations of cells with stem cell-like properties, termed cancer stem cells (CSCs), which are the cause of tumor development, therapy resistance, tumor recurrence and metastasis [7]. Apart from self-renewal and generation of well-differentiated progeny, these cells are also highly tumorigenic and resistant toward conventional cancer treatment regimens [8]. Radio- and chemoresistance is due to intrinsic or acquired mechanisms, such as senescence, increased DNA repair capacity, overexpression of ATP-binding cassette (ABC) drug efflux transporters, activation of survival pathways and intracellular scavenging of reactive oxygen species (ROS) [9].

Several cell surface and intracellular markers are essential for the identification and isolation of CSCs. One of the most used CSC-specific markers is the pentaspan transmembrane glycoprotein CD133, also known as Prominin-1. CD133 was first identified as a hematopoietic stem cell marker and later described as a CSC marker in OC and other solid tumors [10,11]. In OC, CD133 expression correlates with poor prognosis for patients [12].

L1 cell adhesion molecule (L1CAM) is a heavily glycosylated type I transmembrane protein with an essential role in the development of the nervous system and human malignancies [13]. Low expression in healthy tissues facilitated the application of anti-L1CAM monoclonal antibodies (mAbs) for targeted OC therapy [14,15]. This protein displays static and motility-promoting function, controlling cell adhesion and driving migration during neural development [16]. L1CAM in cancer promotes motility and invasiveness, supporting aggressive tumor growth, metastasis and chemoresistance and in many human cancers, such as ovarian and endometrial carcinoma, frequently correlates to poor prognosis and advanced tumor stage [13].

While the precise mechanisms behind OC chemoresistance and metastasis are not yet fully understood, recent studies highlighted a tight connection between epithelial-to-mesenchymal transition (EMT) and CSCs in the context of drug resistance, tumor relapse and metastasis of OC patients [17]. With the activation of EMT, cancer cells gain migratory and invasive properties throughout cancer progression. Metastatic tumor cells lose the expression of epithelial markers such as the cell adhesion molecule E-cadherin and shift towards a mesenchymal phenotype defined by the expression of mesenchymal markers, including vimentin, and the activation of EMT inducing transcription factors [18,19]. In several different types of cancers, including OC, EMT seems to be relevant to the acquirement and maintenance of stem cell-like characteristics [20] and high levels of L1CAM have been associated with EMT [21].

In this study, we aimed to elucidate the role of L1CAM as a marker for ovarian CSCs. Our results suggest that L1CAM expression correlates with intermediate EMT phenotype and, in combination with CD133, defines a specific population of ovarian CSCs with increased radioresistance, enhanced spherogenic and clonogenic property, high tumor take rate, self-renewal capacity and fast tumor growth in vivo. L1CAM was found on all CD133+ OC cells and is responsible for the radioresistance of these cells.

## 2. Results

### 2.1. L1CAM+/CD133+ Ovarian Cancer Cells Show the Highest Clonogenic and Spherogenic Properties as Well as Radioresistance

**Figure 1:**
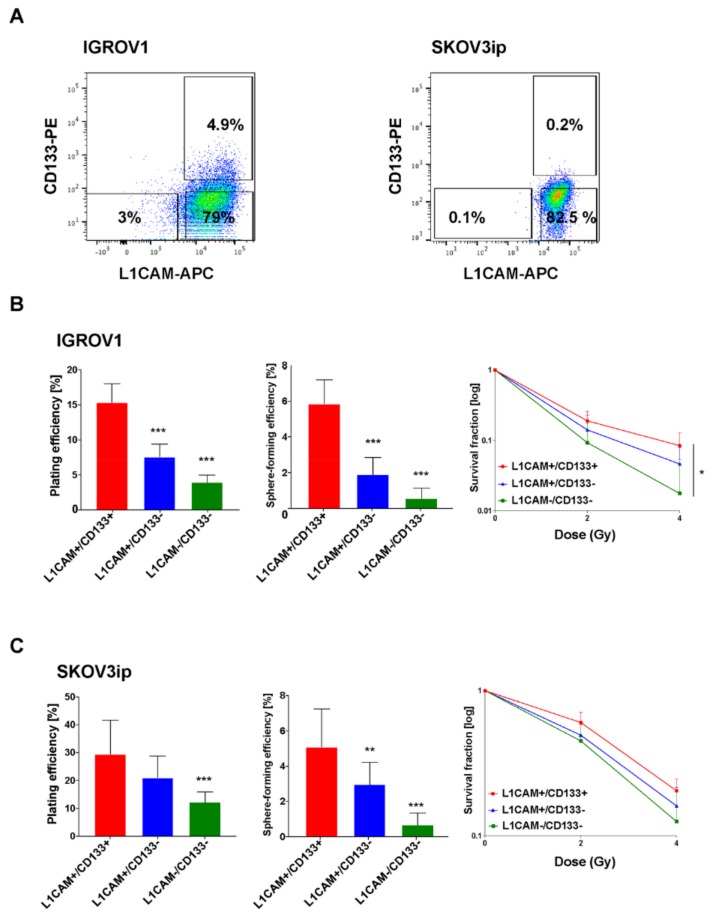

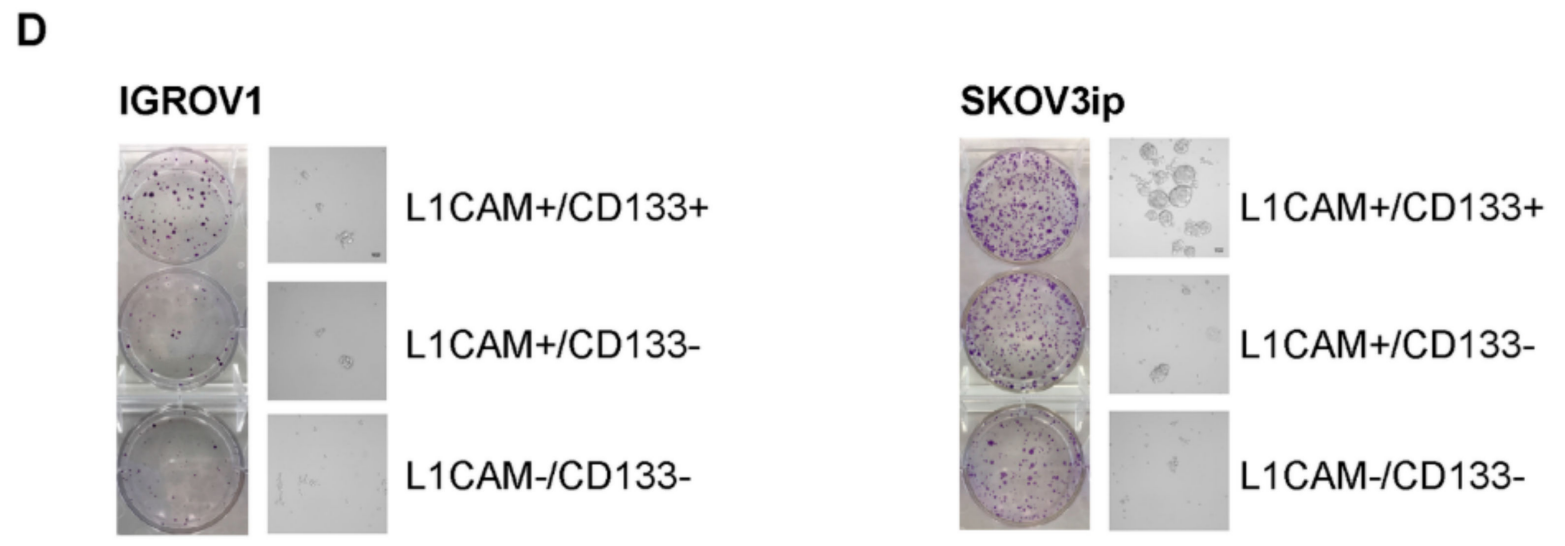
Double-positive L1CAM+/CD133+ cells display enhanced clonogenic and spherogenic properties and radioresistance in IGROV1 and SKOV3ip cells. (**A**) Representative FACS pseudocolor dot plot of IGROV1 (left) and SKOV3ip (right) cells. Gating was performed as exemplified, according to isotype-matched IgG controls. (**B**) Clonogenic capacity (left graph), spherogenic capacity (middle graph) and radiosensitivity (right graph) of IGROV1 cells FACS-sorted for L1CAM and CD133. Each experiment has been performed three times in triplicate and data are expressed as the mean ± SD. One-way and two-way ANOVA; * *p* < 0.03 and *** *p* < 0.001. (**C**) Clonogenic capacity (left graph), spherogenic capacity (middle graph) and radiation responsiveness (right graph) of SKOV3ip cells FACS-sorted for L1CAM and CD133. Each experiment has been performed three times in triplicate and data are expressed as the mean ± SD. One-way and two-way ANOVA; ** *p* < 0.002 and *** *p* < 0.001. (**D**) Representative images of 2D colonies and 3D spheres of IGROV1 (left) and SKOV3ip (right) FACS-sorted cells.

The expression of L1CAM, in combination with CD133, defines a CSC population in glioma [22]. So far, it is unknown whether L1CAM plays a role in ovarian CSCs. We investigated the clonogenic capacity, the clonogenic survival upon irradiation together with the sphere-forming capacity of different OC cell populations defined by the expression of L1CAM and CD133. IGROV1 (p53wt) and SKOV3ip (p53del) cells were stained with fluorescent-labeled antibodies and three different cancer cell populations (double-positive: L1CAM+/CD133+, L1CAM+/CD133−, double-negative: L1CAM−/CD133−) were isolated by fluorescence-activated cell sorting (FACS) (Figure 1A). Of note, in both cell lines no L1CAM−/CD133+ cell population was found.

In the two cell lines, the dual expression of L1CAM and CD133 demonstrated significantly increased clonogenic and spherogenic capacity (Figure 1B,C; *** *p* < 0.001) compared to double-negative cells. Additionally, the 2D radiobiological clonogenic survival assays revealed the highest radioresistance of L1CAM+/CD133+ cell subset (Figure 1B,C; ** *p* < 0.002 for SKOV3ip). In line with previous results [23], SKOV3ip cells formed dense and well-defined spheres while IGROV1 cells formed large and loose aggregates (Figure 1D). Similar results were obtained using the high-grade serous OC cell line Kuramochi (Figure S1). However, due to no tumorigenicity in nude mice [24], we did not use this cell line for in vivo experiments.

### 2.2. L1CAM Triggers Radioresistance in L1CAM+/CD133+ Population

To analyze which role L1CAM plays in the double-positive cells, we generated the L1CAM−/CD133+ cell population (missing in all wild-type cell lines) by means of the genome editing technology CRISPR-Cas9 (Figures S2 and S3).

**Figure 2:**
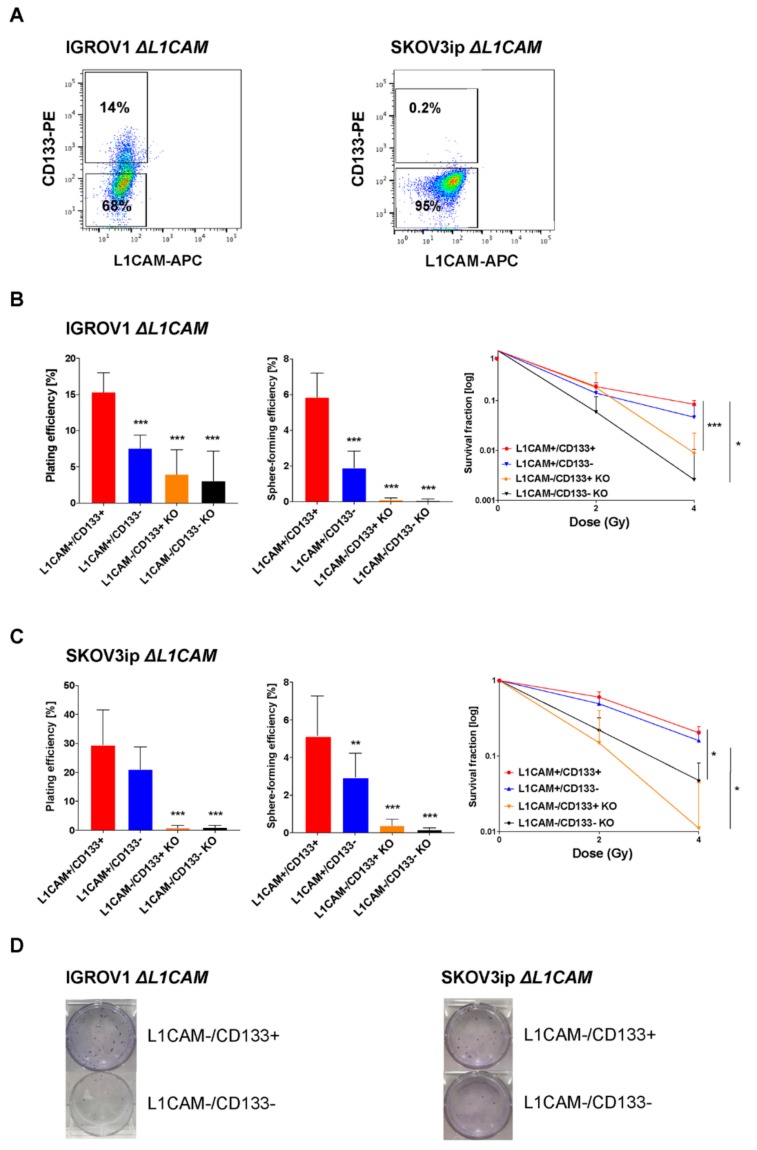
L1CAM triggers radioresistance in L1CAM+/CD133+ IGROV1 and SKOV3ip cells. (**A**) Representative FACS pseudocolor dot plot of IGROV1 ∆L1CAM (left) and SKOV3ip ∆L1CAM (right) cells. Gating was performed as exemplified, according to isotype-matched IgG controls. (**B**) Clonogenic capacity (left graph), spherogenic capacity (middle graph) and radiosensitivity (right graph) of IGROV1 wild-type and ∆L1CAM cells FACS-sorted for L1CAM and CD133. Each experiment has been performed three times in triplicate and data are expressed as the mean ± SD. One-way and two-way ANOVA; * *p* < 0.03 and *** *p* < 0.001. (**C**) Clonogenic capacity (left graph), spherogenic capacity (middle graph) and radiation responsiveness (right graph) of SKOV3ip wild-type and ∆L1CAM cells FACS-sorted for L1CAM and CD133. Each experiment has been performed three times in triplicate and data are expressed as the mean ± SD. One-way and two-way ANOVA; * *p* < 0.03, ** *p* < 0.002 and *** *p* < 0.001. (**D**) Representative images of 2D colonies of IGROV1 ∆L1CAM (left) and SKOV3ip ∆L1CAM (right) FACS-sorted cells.

First, we assessed the contribution of L1CAM on radioresistance in ovarian CSCs. IGROV1 and SKOV3ip ∆L1CAM cell lines wer e used to isolate L1CAM−/CD133+ population by FACS (Figure 2A). FACS analysis revealed an increase in CD133 expression upon L1CAM deletion in IGROV1 (Figure 1A and Figure 2A; IGROV1 wild type 4.9% vs. IGROV1 ∆L1CAM 14%).

Two L1CAM negative cell populations (L1CAM−/CD133+ and L1CAM−/CD133−) isolated from ∆L1CAM IGROV1 and SKOV3ip cells displayed significantly reduced plating efficiency (*** *p* < 0.001), sphere-forming capacity (*** *p* < 0.001) along with radioresistance, in comparison to double-positive cells (Figure 2B–D). The results indicate that the expression of CD133 alone is not sufficient to confer radioresistance and that L1CAM is mainly responsible for radioresistance in the double-positive population. IGROV1 and SKOV3ip ∆L1CAM cells showed significantly decreased clonogenic (*** *p* < 0.001) and spherogenic properties (*** *p* < 0.001) as well as radioresistance in comparison to the bulk population of wild-type cells (Figure S4A–D).

Additionally, ∆L1CAM cells exhibited reduced proliferation rate and reduced migration properties when compared with wild-type cells (Figure S4E–H). Thus, L1CAM contributed to a more stem-like phenotype. To confirm our results, we restored L1CAM expression in ∆L1CAM IGROV1 cells by re-expression of full-length L1CAM using lentiviral transduction. (Figure S3C,D). Here, re-expression of L1CAM in ∆L1CAM cells partially rescued these phenotypes in (Figure S5).

### 2.3. L1CAM+/CD133+ Cells Have Higher Tumor Take, Fast Tumor Growth and Self-Renewal Capacity In Vivo

One of the key properties of CSCs is their ability to form tumors at very high dilutions. In vivo limiting dilution assay (LDA) is a standard technique utilized to estimate frequency of CSCs in immunocompromised mice [25]. Tumor-initiating capacity of SKOV3ip and IGROV1 cells sorted by FACS for L1CAM and CD133 (500, 1000 and 3500) was assessed in CD1 nude mice (Table 1).

SKOV3ip L1CAM+/CD133+ cells showed the highest tumor take in comparison to all other cell populations. Instead, L1CAM−/CD133− cells were not able to form tumors at any dilutions. Using the ELDA (Extreme Limiting Dilution Analysis) software we estimated that the cancer cell-initiating frequency of double-positive (1/1756) was eight times higher in comparison to the one of L1CAM+/CD133− (1/14749) and eight times higher than SKOV3ip wild-type cells (1/13963).

In contrast, in IGROV1, the double-negative cell population displayed high tumorigenicity and long tumor latency (Figure S6). Tumors appeared with median latencies of 36 days (L1CAM+/CD133+), 50 days (L1CAM+/CD133−), 57 days (L1CAM−/CD133+) and 52 days (IGROV1 wild-type bulk). ELDA analysis revealed that the cancer cell-initiating frequency of double-positive (1/316) was four times higher in comparison to the one of L1CAM+/CD133− (1/1245), three times higher than double-negative (1/1056) and six times higher than IGROV1 wild-type cells (1/1881).

After re-analysis of IGROV1 sorted cells, we observed a cell population purity of approximately 60% (Figure S7), demonstrating some cross-contamination with other cell populations. We decided to perform a re-implantation experiment to confirm long-term self-renewal capacity of double-positive cells, which is a major feature of CSCs as they recapitulate the original tumor composition when transplanted several times into animals [26].

Xenograft-derived tumors formed by IGROV1 FACS-sorted cells (Table 1) were collected and cancer cells were enzymatic dissociated with Dispase II and re-injected into a new set of mice (Scheme is shown in Figure S8). Flow cytometric analysis of the dissociated cells before re-injection (Table S2) demonstrated that L1CAM−/CD133− cells have quite stable phenotype (L1CAM: 9.1 ± 1.1%, L1CAM+CD133: 0.9 ± 0.8%). In contrast, L1CAM+/CD133+ cells were able to recapitulate the original cell population compositions (L1CAM: 61.7 ± 1.6%, L1CAM+CD133: 13.3 ± 2.1%). We observed that only double-positive cells were able to regenerate tumors when re-injected in the second round of xenograft, indicating self-renewal property of this cell population (Table 2; eight formed tumors out of eight injected).

**Figure 3:**
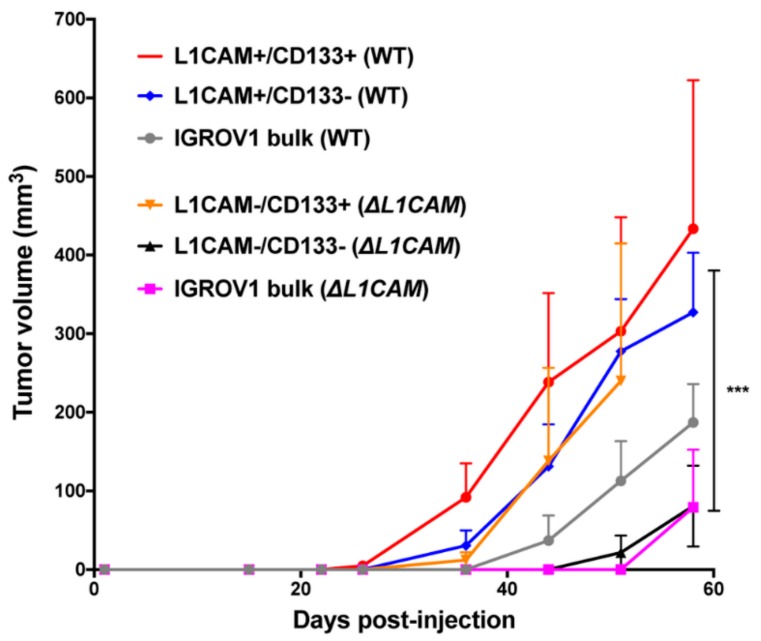
L1CAM+/CD133+ IGROV1 cells show faster tumor growth in vivo. Tumor initiation assay was performed with ovarian cancer wild-type and ∆L1CAM IGROV1 cells isolated by FACS based on L1CAM and CD133 expression. Five hundred cells were subcutaneously injected into CD1 nude mice (n = 6). Tumor volume was measured once a week and the average tumor volume per group ± SEM was calculated. Statistical significance was determined using two-way ANOVA comparing groups on day 58; *** *p* < 0.001. For the significance testing L1CAM−/CD133− vs. L1CAM+/CD133+ cells were compared.

To isolate pure L1CAM− populations, we FACS-sorted ∆L1CAM IGROV1 cells for double-negative, L1CAM−/CD133+ and bulk population. The IGROV1 wild-type cell line was also sorted into double-positive, L1CAM+/CD133− cells and bulk population. Five hundred sorted IGROV1 ∆L1CAM bulk cells formed fewer tumors (4/12) and had longer median tumor latency (58–86 days) in nude mice in comparison to IGROV1 wild-type bulk cells (12/12, 40–89 days) (Table S3). The results demonstrated that IGROV1 L1CAM−/CD133− cells are still able to induce tumors in mice (7/12) but had a significantly smaller tumors volume and longer latency time in comparison to L1CAM+/CD133+ population (Figure 3 and Figure S9). Tumors appeared with median latencies of 40 days (L1CAM+/CD133+ and L1CAM+/CD133−), 47 days (IGROV1 wild type), 86 days (L1CAM−/CD133+), 64 days (L1CAM−/CD133−) and 78 days (IGROV1 ∆L1CAM).

### 2.4. L1CAM Expression Coincides with Epithelial and Intermediate EMT Phenotypes in Ovarian Cancer Cell Lines

During cancer progression, the phenotypic transition from well-differentiated epithelial into a mesenchymal-like state enables cancer cells to disseminate and to metastasize. Growing evidence supports the critical link between EMT and cancer stemness [27].

Therefore, we evaluated the protein expression of L1CAM, vimentin (Vim) as mesenchymal marker and E-cadherin as epithelial marker in 14 OC cell lines by flow cytometry (Table 3 and Figure S10). The OC cell lines used for the screen have been previously classified based on EMT phenotype [28].

With the exceptions of BG1 and OVCAR5, L1CAM was highly expressed (>50%) in all OC cell lines with epithelial (E-cadherin+/Vim−) and intermediate EMT phenotype (E-cadherin+/−, Vim+/−; (Table 3). In contrast, cell lines with a purely mesenchymal phenotype (E-cadherin−/Vim+) displayed a very low or no L1CAM expression. The loss of L1CAM shifts the cells to a more mesenchymal-like state characterized by loss of E-cadherin and claudin-1, markers for epithelial cells (Figure S11A). In line, immunohistochemical analysis of xenograft-derived IGROV1 tumors revealed an increase of vimentin expression in ∆L1CAM tumors compared to wild-type tissues (Figure S11B; wild type: 20–30% vs. ∆L1CAM: 60%).

Additionally, tumors derived from IGROV1 wild-type cells showed constant internalization of E-cadherin (E-cadherinis in the cytoplasm) in contrast to those derived from IGROV1 ∆L1CAM cells, where E-cadherin was expressed as membrane associated protein (Figure S11B).

We also analyzed the expression of putative CSC markers (L1CAM, CD133, CD24, CD44, CD326 and ALDH) in all human OC cell lines (Table S4). With the exceptions of OVCAR4 and OAW42 cells, CD133 and its co-expression with L1CAM were present as small subpopulation of cells in the majority of tested cell lines. All cell lines expressed very high levels (>50%) of at least one of these putative stem cell markers.

### 2.5. L1CAM Regulates the Expression of Stemness and EMT-Associated Genes

**Figure 4:**
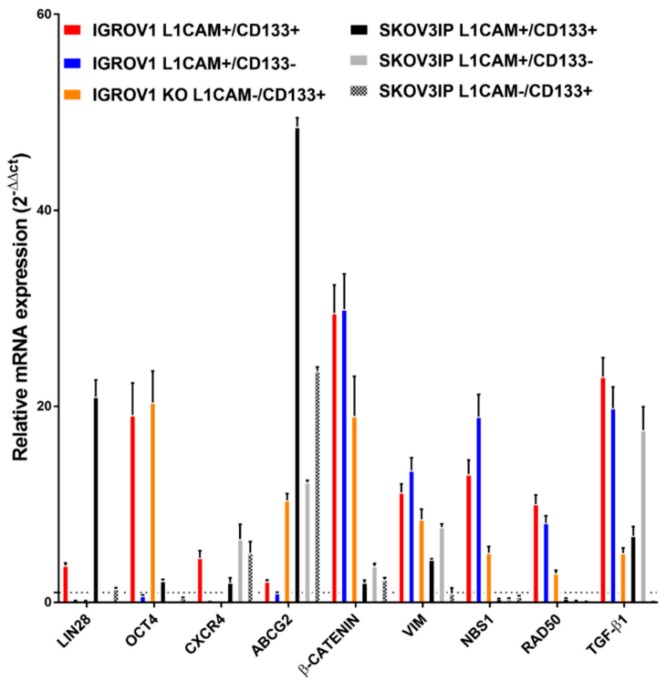
The expression of some cancer stem cell and EMT-related genes is upregulated in IGROV1 and SKOV3ip L1CAM+/CD133+ cells. Fluorescence-activated sorted IGROV1 (left graph) and SKOV3ip (right graph) cell populations were analyzed for the expression of genes related to stemness and EMT transition. Data are expressed as relative mRNA expression (2-ΔΔCT) of reported genes as compared with double-negative cells (dashed line). The experiment has been performed three times in triplicate and data are expressed as the mean ± SD.

To gain insights into the potential molecular mechanisms behind the role of L1CAM in CSC-like traits of ovarian CSCs, we tested the expression of several stem-cell and EMT-related genes known to be up-regulated in cancer stem cell populations in OC and in different other cancer types [29]. Validation by RT-qPCR (Figure 4) indicate that mRNA levels of LIN28, octamer binding transcription factor 4 (OCT4), C-X-C chemokine receptor type 4 (CXCR4), ATP-binding cassette super-family G member 2 (ABCG2), β-catenin, vimentin and transforming growth factor beta 1 (TGF-β1) are expressed more in the L1CAM+/CD133+ cell population derived from IGROV1 and SKOV3ip, indicating aggressive and stem-like properties of these cells. In addition, we analyzed the association of L1CAM, vimentin and TGF-β1 expression with the overall survival (Kaplan Meier Plot) from a data set of 300 ovarian serous cystadenocarcinoma tumor samples (The Cancer Genom Atlas, TCGA RNAseq expression, Pan-Cancer Atlas; Figure S12). We found that L1CAM, vimentin and TGF-β1 expression has a strong tendency toward co-occurrence in tumor tissues, and high expression of this gene signature is associated with reduced overall survival.

### 2.6. Human Ovarian Cancer Ascites Samples and Ovarian Cancer Cell Lines Display Heterogeneous Expression of Different Cell-Surface Markers

In order to translate our in vitro and in vivo data into the human situation, we analyzed a total of 12 advanced-stage OC patient ascites-derived cells for the expression of L1CAM, CD133, L1CAM + CD133, CD24, CD44, CD326 and ALDH using flow cytometry (Table S5 and Figure S13). Clinicopathological data of patients are shown in Table S6.

We found detectable L1CAM expression in all ascites specimens with different numbers of antigen-expressing cells (2.1% to 94.9%). Similar to the FACS-based screening in OC cell lines, we observed generally high percentage (>94%) of CD44 expressing cells in all ascites-derived cells tested. With the exception of patient Nr. 4, the expression of CD133 alone and in combination with L1CAM was quite low in all ascites tested (CD133: 0% to 1.8%, L1CAM+/CD133+: 0% to 1.5%). The expression of the other markers (CD24, CD36 and ALDH) was highly heterogeneous among patients.

## 3. Discussion

CSCs are known to have high clonogenic capacity and to be chemo- and radioresistant towards standard cancer therapies. It is now clear that in the same type of tumor, there are different populations of CSCs defined by one specific marker or different combinations of markers. None of the CSC markers identified so far are exclusively expressed in cancer tissues and for this reason, it is crucial to use a combination of markers to specifically detect CSCs.

In the present study, we identify L1CAM, in combination with CD133, as a new ovarian CSC marker. Bao and colleagues revealed that these two markers define a glioma CSC population, indicating L1CAM as a CSC-specific therapeutic target [22]. We could demonstrate in three OC cell lines (IGROV1, SKOV3ip, and high-grade serous Kuramochi) that a cell population expressing both L1CAM and CD133 has higher clonogenic and spherogenic capacity and is more resistant towards ionizing radiation. Tumor spheres contain high amounts of cells with CSC-like characteristics, such as upregulation of stemness-related genes, self-renewal, high proliferation and differentiation potential [30]. For this reason, spheres display highly aggressive phenotype in growth, migration and invasion and have high resistance to chemotherapeutic drugs in vitro [31]. There are also data demonstrating that sphere-forming cells found in ascites are tumorigenic in vivo and therefore may play a role in metastatic disease [32].

Since we could not find the L1CAM−/CD133+ cell population in all three wild-type cell lines, we decided to create L1CAM knockout cell lines using the innovative genome-editing tool CRISPR-Cas9. L1CAM was deleted in IGROV1 and SKOV3ip cells. The L1CAM knockout cells displayed reduced radioresistance, plating efficiency, sphere-forming capacity and proliferation rate in comparison to the parental cell lines. We also observed that the expression of CD133 alone was not sufficient to confer radioresistance. This finding suggests that L1CAM is mainly responsible for radioresistance in L1CAM+/CD133+ cells.

Consistent with the CSC hypothesis, dual expression of L1CAM and CD133 is present as a small percentage in the majority of OC cells. We provided in vivo evidence that small L1CAM+/CD133+ cell populations are capable of tumor initiation with fast tumor growth. Importantly, when isolated and re-injected into mice, these cells are able to form tumors with the same original tumor cell composition. We detected tumor growth of L1CAM−/CD133− IGROV1 tumor cells, which can be explained by (i) the existence of additional tumor-initiating populations defined by different markers or (ii) cross-contamination with other cell populations that can potentially occur during cell sorting. Epithelial-to-mesenchymal transition (EMT) is induced and regulated by multiple signals and plays a significant role in cancer progression, tumor recurrence, metastatic process and chemoresistance. In OC, the EMT program has been associated with a highly invasive phenotype in the peritoneal cavity [33].

Our results demonstrated that L1CAM expression correlates with intermediate EMT phenotype, stem cell-like state and radioresistance. In IGROV1 cell line the loss of L1CAM shifted the cells to a more mesenchymal phenotype and immunohistochemistry results showed that L1CAM expression is associated with internalization of E-cadherin. Many tumor cells lose epithelial phenotype through protein internalization, leading to an intermediate EMT phenotype [34]. Our results also demonstrated that CD133 expression increased in IGROV1 ∆L1CAM cells. In lung adenocarcinoma, elevated expression CD133 is a signature marker of EMT [35]. Recent studies have highlighted a link between EMT and CSC formation, indicating that stem cell-properties emerge during progression of EMT and stemness seems to be important to regulate invasive potential [36]. Many CSCs isolated from carcinomas possess typical characteristics of cells that have undergone EMT [37]. Remarkably, a recent publication revealed that antibody therapy against human L1CAM in transgenic mouse model induces EMT [38]. Lupia and colleagues recently showed that the expression of CD73 promotes stemness and EMT in ovarian CSCs [39].

In 2011, Strauss et al. identified subpopulations of cells in an intermediate EMT hybrid stage, i.e., cells that retain epithelial markers (i.e., cell-adhesion molecules) but start to gain migratory and invasive properties, switching to mesenchymal expression [40]. These data illustrate that these cells have self-renewal capacity and, under some conditions, are able to differentiate into different lineages. These findings suggest that cells in an “intermediate state” might be more flexible in regulating cell invasion and stem-like properties.

To find an additional link between L1CAM expression and stemness, we tested the expression of several CSC- and EMT-related genes known to be up-regulated in ovarian CSC populations. We selected LIN28 and OCT4, which are transcription factors involved in self-renewal and pluripotency in embryonic stem cells and their co-expression has been associated with ovarian CSCs [41]. CXCR4 is chemokine receptor involved in survival and proliferation [42]. The expression of ABCG2 as an ATP-binding cassette (ABC) transporter correlates with cisplatin and paclitaxel resistance in several OC cell lines, and it has been used to identify CSCs [43]. The canonical Wnt/β-catenin signaling pathway is involved in modulating self-renewal in normal tissues but in OC plays a role in sustaining stem cells and chemoresistance [44]. Previous publications demonstrated the regulation of L1CAM through the Wnt/β-catenin signaling and nuclear β-catenin appears to co-localize with L1CAM in tumor sections [13]. Vimentin is a marker commonly employed for mesenchymal cells or cells undergoing EMT, but it was also found to be a marker of poor chemoresponse in metastatic ovarian carcinoma [45]. TGF-β1 acts as a potent driver of cancer progression through the induction of EMT [46]. Considering the interplay between EMT and stemness, TGF-β1 signaling pathway has been linked to the induction of ovarian CSCs [47]. In breast and pancreatic cancer, TGF-β1 was shown to induce an EMT-like phenotype and leads to expression of L1CAM [21]. We observed that all these genes are overexpressed in the L1CAM+/CD133+ cell population derived from IGROV1 and SKOV3ip, indicating aggressive and stem-like properties of these cells. Previous studies reported that TGF-β-mediated ETM regulates the expression of vimentin, supporting tumor aggressiveness [48,49]. In particular, the expression of vimentin and TGF-β has been linked to poor disease-survival for several cancer patients [50,51]. In line with these data, we found that L1CAM, vimentin and TGF-β1 are co-regulated in ovarian serous cystadenocarcinoma, and high expression of this gene signature is associated with reduced overall survival in a cohort of patients with ovarian serous cystadenocarcinoma. Taken together, this study suggests that L1CAM, vimentin and TGF-β1 may represent a subgroup with aggressive tumors and a high change of recurrence. Future research is needed to validate our findings by using additional L1CAM knockout cell cultures, by functional characterization of L1CAM cells from primary ovarian carcinoma cells, by mechanistic analysis of L1CAM-mediated regulation of cell stemness and radioresistance, as well as by validation of L1CAM expression levels as prognostic factors of the clinical outcome of ovarian carcinoma patients.

## 4. Materials and Methods

### 4.1. Cell Culture

All ovarian cancer cell lines and HEK293T cells were cultivated in RPMI 1640 medium. The media were supplemented with 10% FCS, 100 U/mL penicillin and 100 μg/mL streptomycin. All cell lines were authenticated using STR profiling and regularly tested for the absence of mycoplasma. Patient-derived cells were cultivated for 3–4 passages in 1:1 DMEM/Ham’s F-12 supplemented with 10% FCS. Cell lines were maintained at 37 °C in 95% humidified atmosphere containing 5% CO_2_. Media and additives were obtained from Sigma-Aldrich (Buchs, Switzerland) if not otherwise specified. Ovarian cancer cell lines and patient-derived cancer cells were kindly provided by Viola Heinzelmann-Schwarz (ethical approval EKNZ 2017-01900). SKOV3ip cells were kindly provided by P. Altevogt (German Cancer Research Center, Heidelberg, Germany).

### 4.2. Flow Cytometry and Fluorescence-Activated Cell Sorting (FACS)

Cells were washed once with PBS, detached with Accutase (STEMCELL Technologies, Grenoble, France) and adjusted to final concentration in FACS Buffer (3% FCS in PBS). Samples were examined on a Guava EasyCyte Flow Cytometer (Merck Millipore, Schaffausen, Switzerland) or a CytoFLEX flow cytometer (Beckman Coulter, IN, USA) or sorted on a BD FACS Aria Cell Sorter (BD Biosciences, San Jose CA, USA). The data were analyzed with FlowJo (Tree Star, Ashland, OR, USA, version 10). Immunostaining and sorting were performed as described in Supplemental information.

### 4.3. Clonogenic Assay

Cultured cells were seeded with a density of 500 cells/well in 6-well plates and incubated for 10–14 days at 37 °C with 5% CO_2_. The cells were washed once with PBS and simultaneously fixed and stained with 0.05% crystal violet (Sigma-Aldrich, Buchs, Switzerland) in 4% paraformaldehyde. The plates were washed two times with water and dried at room temperature. Colonies with a minimal size of at least 50 cells were manually counted and plating efficiency was calculated as previously described [52].

### 4.4. Radiation Responsiveness Assay

Eighteen hours after plating, the cells were irradiated with doses of 2, 4 and 6 Gy using an external beam (Gammacell 40 Exactor, Best Theratronics, ON, Canada). After 10–14 days of culturing, the colonies were washed once with PBS and simultaneously fixed and stained with 0.05% crystal violet (Sigma-Aldrich, Buchs, Switzerland) in 4% paraformaldehyde. Colonies with at least 50 cells were manually counted. The results of the assays were calculated as surviving fraction:

SF = (counted colonies)/(seeded cells × PE) × 100

PE: plating efficiency.

### 4.5. Sphere-Forming Assay

After sorting, cells were suspended in 1 mL MEBM (Lonza-Cloetics, Basel, Switzerland) supplemented with B27 (Thermo Fisher Scientific—Gibco, Reinach, Switzerland), 20 ng/mL rhEGF (Promega, Dübendorf, Switzerland), 20 ng/mL bFGF (Gibco, Thermo Fisher Scientific, Reinach, Switzerland), rhInsulin (Amimed, BioConcept, Allschwill, Switzerland), 100 U/mL penicillin and 100 µg/mL streptomycin. Cells were seeded in ultra-low attachment 24-well plates (Corning^®^ Costar, Sigma-Aldrich, Buchs, Switzerland) with a density of 1000 cells/mL. After 14 days, spheres with a diameter of at least 50 nm were manually counted.

### 4.6. CRISPR-Cas9-Mediated Depletion of L1CAM

A detailed description of the CRISPR-Cas9 design, molecular cloning, cell-sorting strategy and characterization of homozygously deleted cells is provided in Supplemental information. Design and experimental strategy have been performed as recently described [19,53].

### 4.7. Quantitative Reverse Transcription PCR (RT-qPCR)

RT-qPCR was performed using L1CAM, LIN28, OCT4, CXCR4, ABCG2, β-CATENIN, VIMENTIN, TGF-β1, NBS1, RAD50 and reference gene GAPDH in 10 μL reaction containing 10 ng cDNA (initial total RNA), 200 nM forward and reverse primer, nuclease free water and 1× GoTaq qPCR Master mix on a ViiA™ 7 Real-Time PCR System (Applied Biosystems, Thermo Fisher Scientific, Reinach, Switzerland). All primers are listed in Table S1. RT-qPCR was performed in triplicate and analyzed as recently described [54].

### 4.8. Human Xenograft Model

Animals were housed at the Paul Scherrer Institute, Villigen, and protocols were approved by the cantonal committee on animal experiments and permitted by the responsible cantonal authorities (permission number 75,666/2c, Kanton Aargau). The studies were conducted in compliance with the Swiss laws on animal protection. Limiting dilution assay (LDA) was performed as described in Supplemental information.

### 4.9. Statistical Analysis

Statistical analysis was performed with GraphPad Prism 7 (GraphPad Software, Inc., La Jolla, CA, USA). All experiments were performed in triplicate. Spherogenic, clonogenic and migration assays were analyzed by one-way ANOVA and student’s *t*-test (two-tailed, unpaired). *p* values of < 0.05 were considered statistically significant (*** *p* < 0.001; ** *p* < 0.002; * *p* < 0.03). The differences between cell survival curves were analyzed using the statistical package for the social sciences (SPSS) v23 software as described by Franken and colleagues [52] by fitting the data into the linear-quadratic formula S(D)/S(0) = exp (αD + βD^2^) using stratified linear regression.

## 5. Conclusions

Overall, our results suggested that L1CAM orchestrates ovarian CSC function through the expression of genes that play a key role in stemness and in EMT. Targeting ovarian CSCs with anti-L1CAM antibody offers a new strategy for CSC therapy. In particular, because L1CAM is expressed in all CD133+ ovarian CSCs and, so far, L1CAM has not been described in normal stem cells. In addition, L1CAM is present in the bulk population of ovarian cancer cells.

Our group and others already demonstrated that L1CAM is a promising molecular target for antibody-based therapy [14,15,55,56]. We are now focusing our research on the establishment of effective anti-L1CAM treatment modalities to eradicate ovarian CSCs. Furthermore, characterization of additional L1CAM knockout cell lines, analysis of potential co-expression of L1CAM and CSC markers in the large numbers of OC patients’ samples as well as a combination of L1CAM-targeted treatment with radiotherapy in OC xenograft models are warranted to further validate our findings.

## Figures and Tables

**Table 1:** Tumor take of SKOV3ip and IGROV1 FACS-sorted cells in nude mice.

		DPOS	L1CAM+/CD133−	DNEG	Bulk
SKOV3ip	Dilution 1	2/3 (73–101)	2/6 * (66–108)	0/6 *	1/6 * (59)
	Dilution 2	3/3 (31–59)	0/6 *	0/6 *	0/6 *
	Dilution 3	1/3 (44)	0/6 *	0/6 *	1/6 * (59)
IGROV1	Dilution 1	6/6 (29–79)	4/6 (36–82)	4/6 (36–79)	4/6 (43–149)
	Dilution 2	5/6 (36–64)	5/6 (36–124)	4/6 (50–57)	2/6 (33–85)
	Dilution 3	6/6 (29)	4/6 (36–49)	5/6 (71–96)	4/6 (33–64)

Tumor initiation assay in CD1 nude mice (n = 3) was performed with SKO3ip and IGROV1 cells FACS-sorted for L1CAM and CD133. The bulk population of wild-type cells was used as control. Different dilutions of cells (Dilution 1: 500 cells, Dilution 2: 1000 cells and Dilution 3: 3500 cells) were subcutaneously injected into the mice. Tumor take was determined as number of mice with palpable tumors at day 153 (SKOV3ip) and 155 (IGROV1). DPOS: L1CAM+/CD133+, DNEG: L1CAM−/CD133−. * The numbers indicate how many tumors formed out of the injected ones. The numbers in parenthesis indicate tumor latency in days.

**Table 2:** L1CAM+/CD133+ IGROV1 cells show self-renewal in vivo.

L1CAM+/CD133+	L1CAM+/CD133−	L1CAM−/CD133−	Bulk Population
8/8	0/8	0/4	0/4

Tumor re-implantation assay was performed with OC cells isolated from FACS-sorted IGROV1-induced tumors. Tumors were enzymatic dissociated and two dilutions of cells (500 and 1000) were subcutaneously injected into CD1 nude mice (n = 2–4). Tumor take was determined as number of mice with palpable tumors at day 152.

**Table 3:** L1CAM expression correlates with epithelial and intermediate EMT phenotype in ovarian cancer cell lines.

Cell Line	L1CAM [%]	EMT Class	E-Cadherin	Vimentin
OVCAR4	98.2 ± 1.8	E	+++	−
OVCAR5	19.8 ± 4.2	E	+++	−
CAOV3	99.3 ± 1	E	++	−
OVSAHO	98.6 ± 1.2	E	+++	−
BG1	9.1 ± 5.3	E	++	+
OAW42	98.9 ± 1.1	IE	++	+
IGROV1	99.9 ± 0.1	IE	++	−
SKOV3ip	88.2 ± 0.1	IE	++	+
OVCAR8	91.4 ± 8.5	IM	−	n.d.
Kuramochi	81 ± 5.4	IM	+	++
EFO27	98.6 ± 1.4	M	−	++
A2780	0.3 ± 0.1	M	−	++
TYK-nu	0.8 ± 0.3	M	−	++
TOV112D	2.3 ± 0.8	M	−	++

The expression of L1CAM was analyzed by flow cytometry in a large panel of ovarian cancer cell lines. The numbers indicate the percentage of antigen-expressing cells in the sample population ± SD of three independent experiments. The OC cell lines used for the screen have been previously classified based on EMT phenotype [27]. EMT: epithelial-mesenchymal transition; E: epithelial phenotype; IE: intermediate epithelial phenotype; IM: intermediate mesenchymal phenotype; M: mesenchymal phenotype; +++: high- ; ++: moderate-; +: low expression; n.d.: not defined.

## Supplementary Materials

The following are available online at https://www.mdpi.com/2072-6694/12/1/217/s1. Figure S1: Double-positive L1CAM+/CD133+ cells exhibited enhanced clonogenic and spherogenic properties and radioresistance in Kuramochi cells, Figure S2: Generation of stable and site-specific L1CAM mutant ovarian cancer cell lines using CRISPR-Cas9 technology, Figure S3: Evaluation of CRISPR-Cas9 genome edited IGROV1 and SKOV3ip clones for depletion of L1CAM, Figure S4: IGROV1 and SKOV3ip ∆L1CAM cells showed reduced clonogenic properties, spherogenic capacity, radioresistance, proliferation rate and migration ability and in comparison to wild-type cells, Figure S5: Constitutive L1CAM expression in IGROV1 ∆L1CAM cells partially restored clonogenic properties, spherogenic capacity, radioresistance, migration ability and proliferation as compared to wild-type cells, Figure S6: IGROV1 L1CAM+/CD133+ population showed shorter tumor latency in vivo, Figure S7: Re-analysis of IGROV1 cells isolated by FACS for in vivo limiting dilution assay (LDA), Figure S8: Scheme of the re-implantation experiment, Figure S9: IGROV1 L1CAM−/CD133− cells showed significantly longer tumor latency in vivo compared to L1CAM+/CD133+ population, Figure S10: L1CAM expression in a panel of ovarian cancer cell lines classified based on EMT phenotype, Figure S11: The loss of L1CAM shifted the cells towards a more mesenchymal-like state characterized by loss of E-cadherin and claudin-1, Figure S12: Analysis of the TCGA (PanCancer Atlas, RNAseq expression) dataset, Figure S13: Expression of putative cancer stem cell markers in human ovarian cancer ascites samples is heterogeneous, Table S1: Table provides details of oligonucleotides used in this study, Table S2: Expression of putative cancer stem cell markers of IGROV1 tumor cells before re-injection into nude mice, Table S3: Tumor-initiating capacity of L1CAM/CD133 cell populations isolated from IGROV1 wild-type and ΔL1CAM cell lines, Table S4: Expression of putative cancer stem cell markers in ovarian cancer cell lines, Table S5: Human cancer cells derived from ovarian cancer patients’ ascites and ovarian cancer cell lines show heterogeneous expression of different cell surface markers, and Table S6: Clinicopathological data of ovarian cancer patients.
