# Supplementary material for: L1 Cell Adhesion Molecule Confers Radioresistance to Ovarian Cancer and Defines a New Cancer Stem Cell Population
Source: Cancers (Basel). 2020 Jan 15;12(1):217. doi: 10.3390/cancers12010217 (PMC7017143; doi:10.3390/cancers12010217)
Supplement: Supplementary file 1 [file cancers-12-00217-s001.pdf]

SUPPLEMENTAL INFORMATION

Reagents and antibodies: Chimeric monoclonal antibody chCE7 (human κ light chain and human γ 1 heavy chain) of the IgG1-subtype specific for human L1CAM was produced in HEK293 cell line and purified from cell culture supernatant using protein-A Sepharose column (GE Healthcare, Glattbrug, Switzerland) as previously described by Grünberg et al. [1]. An unspecific isotype-matched IgG was used as a control for experiments. Primary antibodies E-Cadherin (#3195), Tubulin (#2148), claudin-1 (#13255) and secondary antibodies HRP-anti-rabbit IgG (#7074) and HRP-anti-mouse IgG (#7076) were all obtained from Cell Signaling Technology (BioConcept Ltd, Allschwil, Switzerland). Primary antibody GAPDH (#47724) and vimentin (#MA5-11883) secondary antibody HRP-anti-human IgG (#W4038) were obtained from Santa Cruz Biotechnolgy (Heidelberg, Germany), Invitrogen (Thermo Fisher Scientific, Reinach, Switzerland) and Promega (Dübendorf, Switzerland), respectively. Human IgG isotype control (#02-7102) was obtained from Invitrogen (Thermo Fisher Scientific, Reinach, Switzerland). Anti-human CD44-PE (#130-110-293), CD24-PE (#130-095-953), CD133/1-PE (#130-113-108), CD326-PE (#130-113-108) and mouse IgG1 (#130-092-212) antibodies were obtained from Miltenyi Biotec (Bergisch Gladbach, Germany). Secondary antibody Dylight650 (#98593) was purchased from Abcam (Cambridge, UK). Supplements for sphere-forming assay; B27 (#17504044) and bFGF (#13256029) obtained from Gibco (Thermo Fisher Scientific, Reinach, Switzerland), rhEGF (#G5021) obtained from Promega (Dübendorf, Switzerland) and rhInsulin (#5-79F00-G) purchased from Amimed (BioConcept, Allschwill, Switzerland). Ultra-low attachment 24-well plates (#CLS3473-24EA) were obtained from Corning Costar (Sigma-Aldrich, Buchs, Switzerland).

Flow cytomery: Cells were decorated with anti-L1CAM monoclonal antibody chCE7 (50 ng/µl; Paul Scherrer Institute, Villigen, Switzerland) or with purified human IgG1 specific isotype (50 ng/µl; Invitrogen, Reinach, Switzerland) as negative control. Samples were incubated for 30 min in the dark on a shaking platform. After incubation the samples were washed three times with FACS buffer. Thereafter the samples were stained with Dylight650-labeled secondary antibody anti-human IgG goat polyclonal antibody (1:100 dilution; Abcam, Cambridge, UK) in dark for 30 min on a shaking platform. After incubation, the cells were washed three times with FACS buffer or ALDEFLUOR assay buffer and resuspended in 100 µl of corresponding buffer. Alternatively, the cells were stained with PE-labeled anti-CD24, -CD44, -CD133/1 and -CD326 antibodies (dilution according to manufacturer's recommendation; Miltenyi Biotec, Bergisch Gladbach, Germany) in dark for 30 minutes on a shaking platform. After incubation, the cells were washed three times with FACS buffer and resuspended in 100 µL of corresponding buffer. An unspecific isotype-matched IgG was used as a control for each experiment. ALDEFLUOR assay to detect ALDH activity was performed according to the manufacturer's instructions provided in the Aldehyde Dehydrogenase–Based Cell Detection Kit (STEMCELL Technologies, Grenoble, France).

Fluorescence-activated cell sorting (FACS): Around 10 x 10^6 cells were double stained with anti-L1CAM monoclonal antibody chCE7 (50 ng/µl; Paul Scherrer Institute, Villigen, Switzerland) and Dylight650-labeled antibody anti-human IgG Goat polyclonal secondary antibody (1:100; Abcam, Cambridge, UK) in combination with phycoerythrin PE-labeled anti-CD133/1 (dilution according to manufacturer's recommendation; Miltenyi Biotec, Bergisch Gladbach, Germany). Dead and apoptotic cells were separated from live cells using DAPI (BD Bioscience). Matching isotype monoclonal antibodies human IgG (50 ng/µl; Invitrogen, Reinach, Switzerland) and mouse IgG1 (dilution

according to manufacturer's recommendation; Miltenyi Biotec, Bergisch Gladbach, Germany) were used as controls. All investigated cell lines were gated individually to exclude debris, followed by single cell gating to remove dead cells and doublets. Subsequently the cells were plated for clonogenic, spherogenic and 2D responsiveness assay (assays are described in details below).

Western blot: Whole cell lysates were obtained from sub-confluent cultures. Cells were lysed for Western blot analysis according to standard laboratory protocols. The protein concentration of cell lysates was determined by BCA Protein Assay Kit (Pierce, Perbio Science, Lausanne, Switzerland). Equal amounts of protein (20 μg) were loaded and separated using SDS-PAGE, followed by blotting onto a polyvinylidene difluoride membrane (Amersham Biosciences, Otelfingen, Switzerland). The membrane was blocked with 5% (w/v) BSA (Sigma-Aldrich, Buchs, Switzerland) in TBST for 1 h and incubated with primary monoclonal antibodies L1CAM (0.5 μg/μl), E-cadherin (1:1000), vimentin (1:2000), claudin-1 (1:1000), α/β-tubulin (1:1000) and GAPDH (1:1000) diluted in 5% (w/v) BSA in TBST at 4°C overnight. Afterwards, membranes were washed three times in TBST and incubated with corresponding HRP-conjugated secondary antibodies (anti-rabbit, -mouse and -human) in 5% BSA in TBST for 3 h at room temperature. After washing with TBST, detection was carried out with the Super Signal West Dura Extended Duration Substrate (Life technologies).

MTT: To identify the proliferation rate cells were seeded at a density of 1500 cells/well in 96-well plates and incubated for 24 h to 168 h. At each time point, MTT dye (Sigma- Aldrich, Buchs, Switzerland) was added at a final concentration of 500 μg/mL and incubated for 3 h. After removal of supernatant, 200 μl of DMSO were added to dissolve the crystals. Optical density (OD, absorbance at 540 nm) was measured with a Synergy H1 Hybrid Reader (Biotek, Basel, Switzerland). The measurement was performed in quadruplets.

Cell migration assay: Sub-confluent cells were counted and 8 x 10^4 to 1 x 10^5 cells were seeded with serum-free media in 12-well plates into the upper chamber of each insert. Incubation at 37°C for 24 h allowed cells to migrate to the chemo-attractant (growth medium containing 10% FCS). After incubation, medium in the interior part of the insert was removed and the insert was dipped in 0.2% crystal violet in 4% paraformaldehyde for 20 min. The insert was intensively washed and non-migrated cell in the interior of the insert were removed using a cotton-tip swab. Five random areas of the inserts were photographed with an Olympus IX81 microscope and cell count was performed.

Single-guided RNA design and vector construction: Single guided RNAs (sgRNA) targeting exon 2 of *L1CAM* were designed using the web tool of the Zhang laboratory (http://crispr.mit.edu) [2]. SgRNA1 and sgRNA2 (Supplementary Table S1) with scores of 85 and 90, respectively, were selected for gene editing of the translation start site of *L1CAM*. Intended oligo pairs encoding 20nt targeted sequences (Supplementary Table S1) with overhangs (both 5' and 3') from BbsI restriction site were ordered, annealed and finally cloned into pSpCas9(BB)-2A-GFP (PX458, Addgene, #48138) via BsbI restriction site using T4-DNA ligase (Promega, Dübendorf, Switzerland). Constructs were transformed into DH5α E. coli strains and sequenced for confirmation of the sgRNA inserted into PX458 by Sanger DNA sequencing using Primer human U6.

Transfection and single-cell sorting: IGROV1 and SKOV3ip cell lines were grown in 6-well plate (3 x 10^5 – 5 x 10^5 cells/well) for 24 h and transiently transfected using TransIT-X2® Dynamic Delivery System (Mirus, Madison, WI, USA) and Viafect transfection reagent (Promega, Dübendorf,

Switzerland) with 2.5 µg of sgRNA encoded in pSpCas9(BB)-2A-GFP (PX458, Addgene, #48138) donor plasmid to generate homozygous *ΔL1CAM* cells. 72 h after transfection cells were washed with PBS, harvested using Accutase (STEMCELL Technologies, Grenoble, France) and resuspended in RPMI 1640 containing 10% FCS. Single-cell sorting was performed on a BD FACS Aria Cell Sorter (BD Bioscience, San Jose CA, USA) sorting *Cas9*-active cell pools for single GFP+ cells into 96-well flat-bottom plates with pre-warmed RPMI 1640 medium containing 10% FCS. Plates were incubated for up to three weeks following transfer to 48-well plates and genomic DNA isolated for genotyping PCR to characterize single cell clones.

Genotyping PCR: Selected clones were characterized to identify homozygous knockout by using three independent PCR primer pairs (Supplementary Table S1). PCR was performed using 2x PCR Super Master Mix (LuBioScience, Luzern, Switzerland), 300 nM primer, 100 ng genomic DNA (gDNA). PCR conditions were 94°C for 2 min, then 34 cycles of 94°C for 20 sec, 59°C for 30 sec, 72°C for 2 min, finished with 1 cycle at 72°C for 5 min. Amplicons were visualized on a 1% agarose gel.

DNA sequencing: PCR products corresponding to genomic modifications were purified and cloned into the pGEM-T Easy Vector System (Promega, Dübendorf, Switzerland) according to the manufacturer's protocol and sequenced using T7 primer (Supplementary Table S1) by Sanger DNA sequencing service from Microsynth AG (Switzerland).

***L1CAM* rescue and cloning of L1CAM constructs:** To rescue the L1CAM in *ΔL1CAM* cells, we utilized full-length L1CAM (pcDNA3-hL1, Addgene, #89411) and we designed primers to amplify the *L1CAM* open reading frame adding a C-terminal HA tag on the C-terminus (Supplementary Table S1). The PCR was performed using Expand™ High Fidelity PCR System (Roche Switzerland), 300 nM forward and reverse primer, 100 ng genomic DNA (gDNA), 10 mM dNTPs and nuclease-free water under following conditions: 94°C for 2 min followed by 10 cycles of 94°C for 15 sec, 58°C for 30 sec, 68°C for 3 min, and 20 cycles of 94°C for 15 sec, 58°C for 30 sec, 68°C for 1 min 30 sec finished with 1 cycle at 68°C for 7 min. Amplicons were visualized on 1% agarose gel and purified by Wizard SV gel and PCR Clean-Up System (Promega, Dübendorf, Switzerland) and sequenced using T7 primer (Supplementary Table S1) by Sanger DNA sequencing service from Microsynth AG (Switzerland). The desired *L1CAM* open reading frame was then introduced into pUltra (Addgene, #24129) via NheI and XbaI cloning procedure for further lentiviral transduction. All plasmids were partly sequenced using EGFP_F primer (Supplementary Table S1) by Sanger DNA sequencing from Microsynth AG (Switzerland).

Lentivirus production and transduction: HEK293T cells were cultured as described above. One day prior to transfection, 4 x 10^6 cells were seeded in a T75 cm^2 flask. Cells were transfected when they reached 70–80% confluence. For each flask, 4 µg of plasmid pUltra (Addgene, #24129) encoding the genes of interest (L1CAM-HA) and 2 µg of pMD2.G (Addgene, #12259) and 2 µg of pCMVR8.74 (Addgene, #22036) were transfected using 24 µl of jetPEI reagent and 1 ml of 150 mM NaCl solution (Polyplus-transfection, Chemie Brunschwig AG, Basel, Switzerland). Media was changed 24 h after transfection. Virus supernatant was collected 48 h later and filtered with a 0.45 µm polyvinylidene fluoride filter (Millipore), and stored at -80°C. IGROV1 *ΔL1CAM* were transduced with pUltra lentiviral supernatant in 2 ml of media in T25 cm^2 flask and selected after three passages by GFP enrichment.

Immunohistochemistry: Biopsy samples of the tumors were immediately put in 10% neutral buffered formalin for 24 h. Formalin-fixed, paraffin-embedded (FFPE) tissue sections (2 µm thickness) were mounted on positively charged slides and dried overnight at 37°C. Drying was followed by the deparaffinisation of the slides with four xylene baths for 5 min each using the Tissue-Tek®Prisma® and Film® (Sysmex,Horgen, Switzerland). For rehydration, a degressive alcohol series using 100% ethanol, 95% ethanol, 70% ethanol, and distilled water was performed. Further Antibody specific protocols were used. For L1CAM immunohistochemical staining underwent an antigen-retrieval pretreatment after rehydration by putting the slides into EDTA-buffer (basic buffer pH 9.0) and then into a pressure cooker for 20 min at 98°C, followed by rinsing with distilled water. Thereafter all the sections were put in TBS wash-buffer 3006 (Dako, Carpinteria, CA, USA). Staining was performed with Dako Autostainer (Agilent Technologies) using antibodies obtained from Abcam (Cambridge, UK): L1CAM (#Ab208155; 1:500), E-cadherin (#Ab40772; 1:150) and from Dako (Carpinteria, CA, USA): vimentin (#M7020; 1:300). The antibodies were diluted in the dilution-buffer S2022 (Dako, Carpinteria, CA, USA). After incubation with the primary antibody, the slides were rinsed with TBS wash-buffer and blocked with peroxidase (peroxidase blocking buffer, Dako S2023) for 10 min at room temperature. Before removing the slides from the Autostainer, they were rinsed with TBS and incubated with DAB Dako K3468 (Dako, Carpinteria, CA, USA) for 10 min at room temperature. Next, the slides were rinsed with TBS and incubated with the EnVision™ + System HRP Rabbit Kit (Dako K4003) for 30 min at room temperature or incubated with the Dako Real™ Detection Kit (Dako K5001, K5003) for 15 min at room temperature. Before removing the slides from the Autostainer, they were rinsed with TBS and incubated with DAB Dako K3468 (Dako, Carpinteria, CA, USA) for 10 min at room temperature. Removing the slides from the Autostainer, they were rinsed with distilled water and counterstained for 2 s in Hematoxylin (modified acc. to Gill II, Merck KGaA, Darmstadt, Germany). Finally, the sections were rinsed with tap water, dehydrated in the Prisma®machine (70% ethanol, 95% ethanol, 100% ethanol and xylene), and covered with the Tissue-Tek®-Film®. The immunohistochemical staining and H&E staining was investigated by a board certified pathologist (C. Krudewig) for the expression of L1CAM in selected groups as well as for the expression of E-Cadherin and vimentin.

Limiting dilution assay (LDA): Tumor cells were isolated by FACS based on L1CAM and CD133 expression, suspended in PBS:ECM gel (1:1; Sigma-Aldrich, Buchs, Switzerland) and implanted in different dilutions (from 500 to 3500 cells) subcutaneously (s.c) into the right and left flanks of CD1 nude mice (Charles River, Sulzfeld, Germany, 5 weeks old). Animals were monitored for 5 months twice a week for weight and tumor growth. Animals were euthanized when the tumors reached the volume of 1 cm^3. Tumors were resected and a portion of each tumor was fixed in 4% formalin for histologic analysis. Tumor volume was calculated using the $(L \times W^2)/2$ formula. Statistical analysis of tumor growth curves was performed using two-way ANOVA. ELDA (Extreme limiting dilution analysis) was performed using the online tool http://bioinf.wehi.edu.au/software/elda/ (26). For *in vivo* serial passaging, single-cell suspensions were obtained using Dispase II (Sigma-Aldrich, Buchs, Switzerland) as previously described (27) and used for re-injection into mice (500 and 1000 dilutions; 2 tumors per mouse).

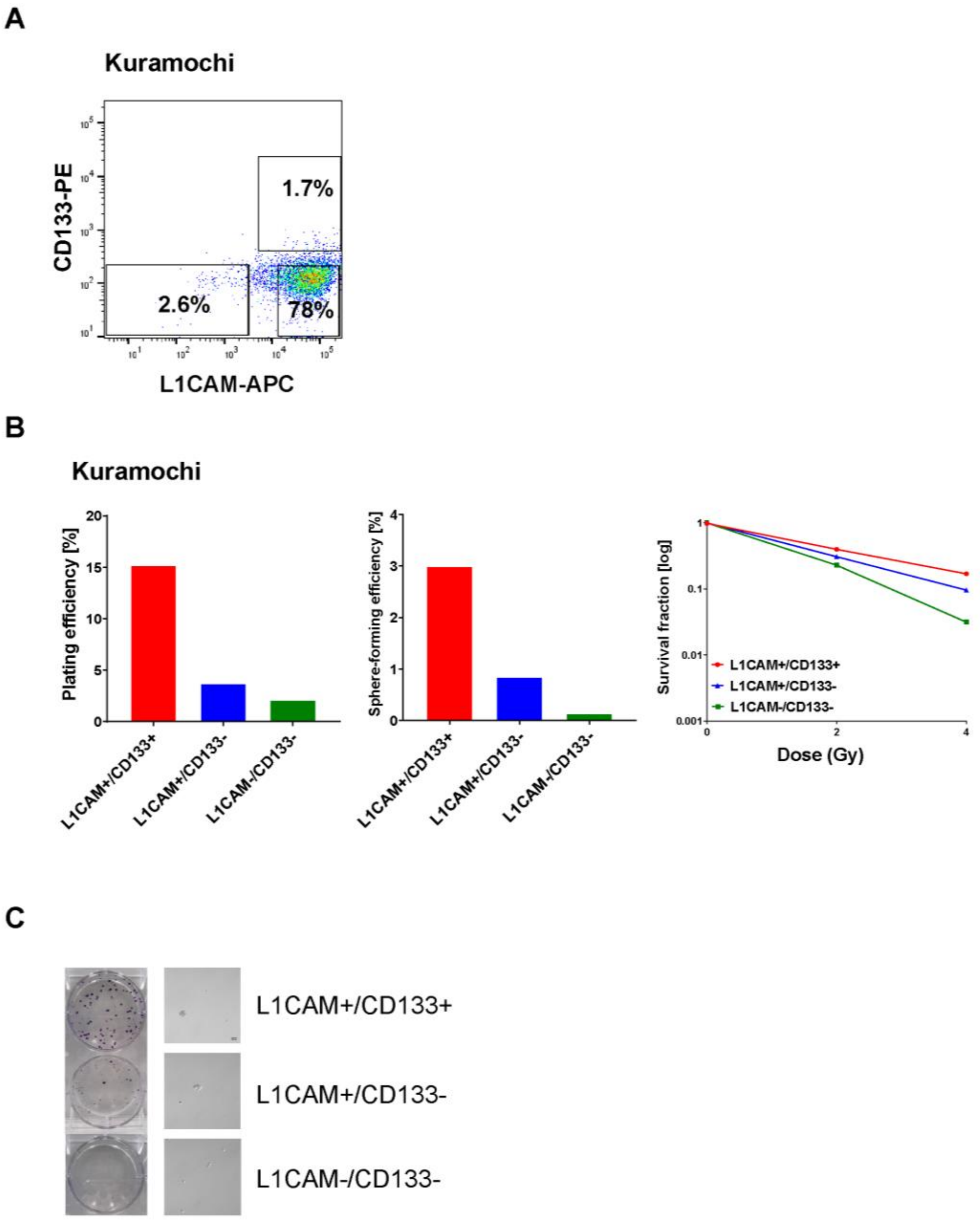

Figure S1. Double positive L1CAM+/CD133+ cells exhibited enhanced clonogenic and spherogenic properties and radioresistance in Kuramochi cells. **(A)** Representative FACS pseudocolor dot plot of Kuramochi cells. Gating was performed as exemplified, accordingly to isotype-matched IgG controls. **(B)** Clonogenic capacity (left graph), spherogenic capacity (middle graph) and radiosensitivity (right graph) of Kuramochi cells FACS-sorted for L1CAM and CD133. The experiment has been performed twice in triplicates. **(C)** Representative images of 2D colonies and 3D spheres of Kuramochi FACS-sorted cells.

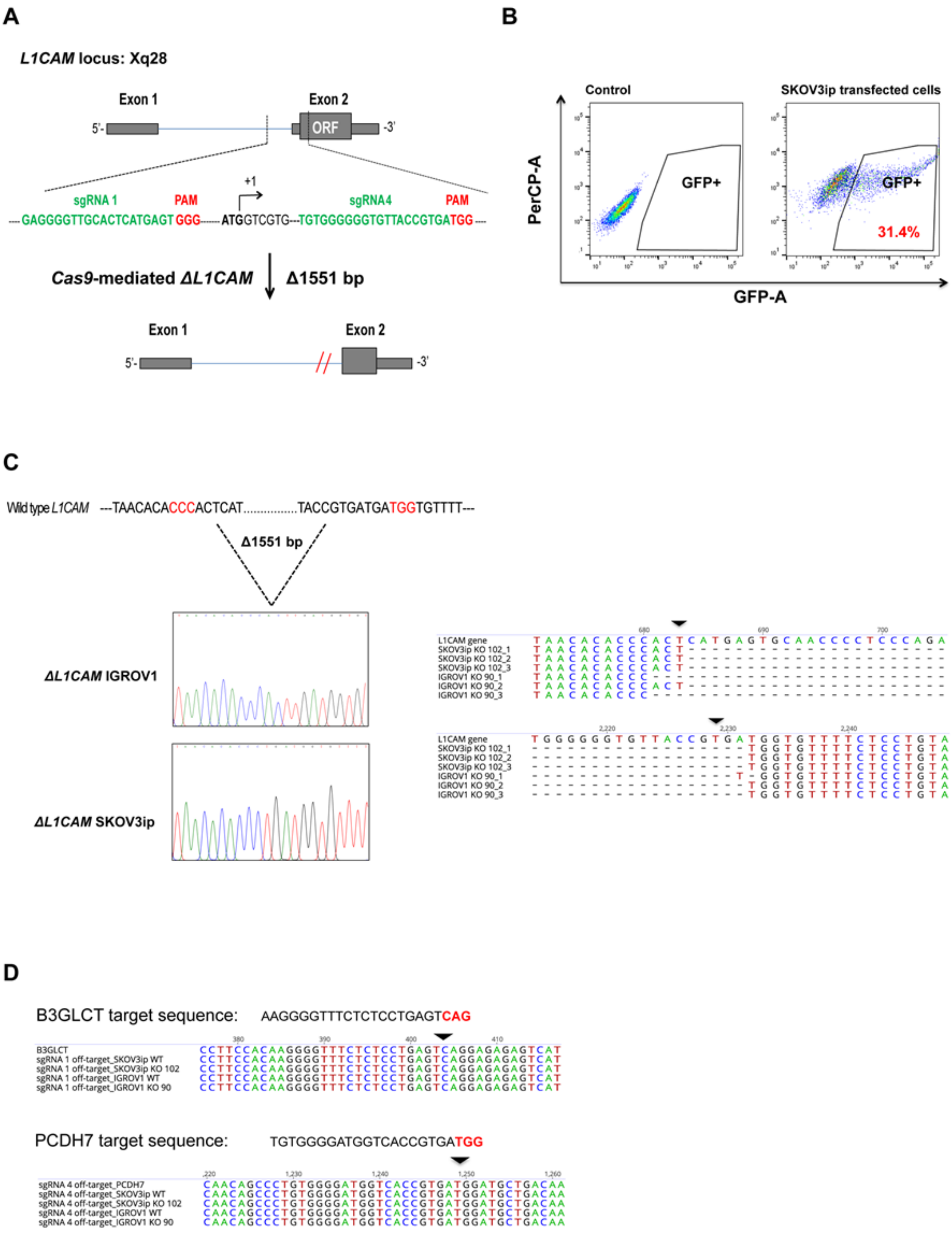

Figure S2. Generation of stable and site-specific L1CAM mutant ovarian cancer cell lines using CRISPR-Cas9 technology. **(A)** General strategy used to delete a genomic locus at the translation start site of the L1CAM gene (ΔL1CAM) using two sgRNAs. **(B)** Single cell sorting strategy after transient transfection of sgRNA CRISPR-Cas9 constructs. Cancer cell lines were transfected with equal amount of PX458 incorporating two different L1CAM-specific sgRNA in addition to Cas9 and GFP. Single cell sorting was performed 48 h after transfection as exemplified for SKOV3ip cells. **(C)** Representative Sanger DNA sequencing of ΔL1CAM clones confirming 1151bp deletion and Sanger DNA sequencing results for selected clones at the Cas9-targeted region showing deletion of L1CAM open reading frame (ORF). **(D)** Sanger DNA sequencing results for validation of possible off-target effects by the two sgRNAs used for genome-editing. No off-target effects were observed at the predicted genomic locus B3GLCT and PCDH7.

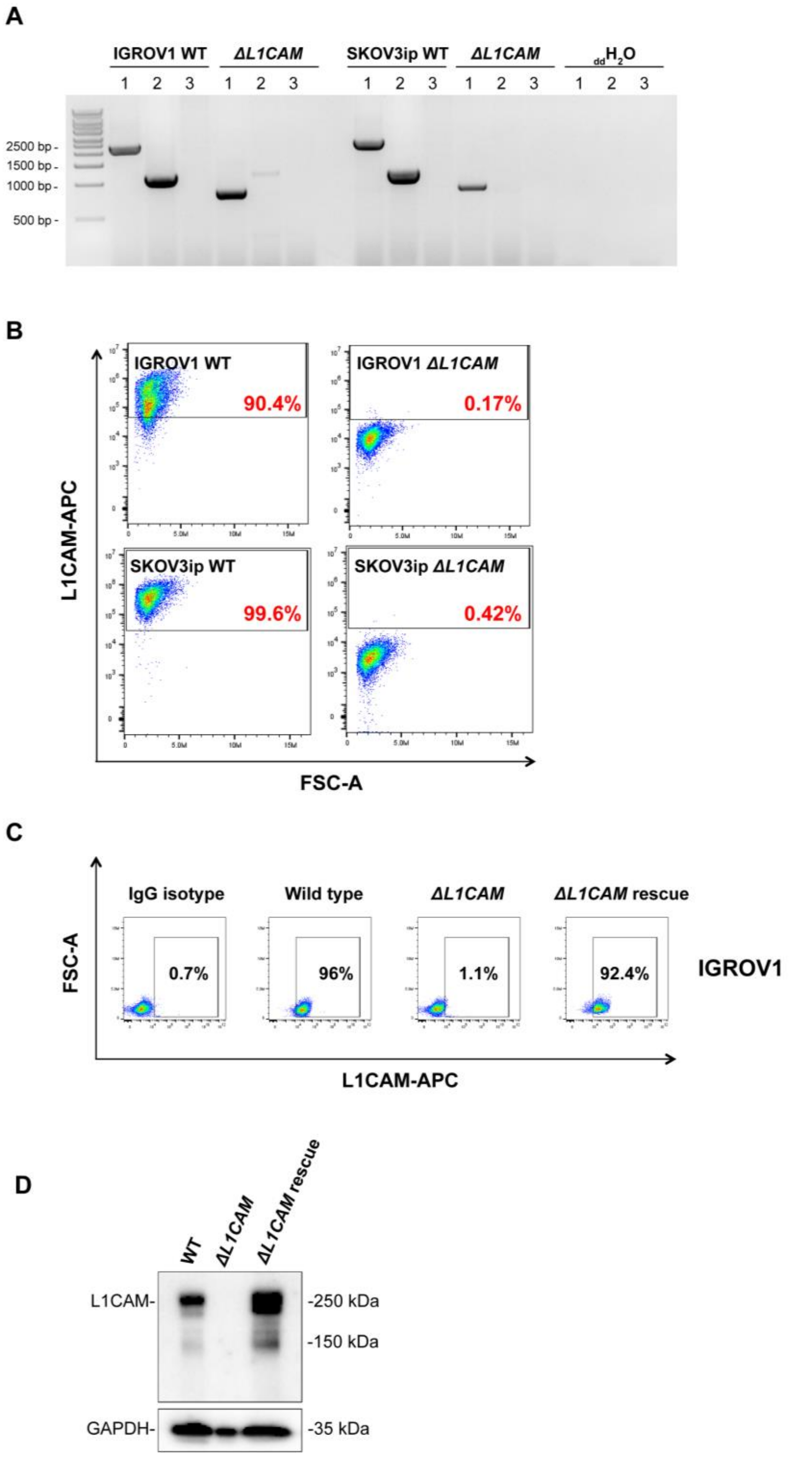

Figure S3. Evaluation of CRISPR-Cas9 genome edited IGROV1 and SKOV3ip clones for depletion of L1CAM. **(A)** Homozygously deleted ΔL1CAM cells harboring a 1551bp deletion were initially identified by genotyping PCR. Selected single cell clones were assayed by three genotyping PCRs (1: Deletion PCR; 2370bp (wild type L1CAM) and 819bp (deletion band), 2: Wild type-specific PCR; 1163bp and 3: Inversion PCR; 1200bp). **(B)** Loss of L1CAM expression in ΔL1CAM cells assessed by flow cytometry **(C)** Corresponding flow cytometry results shown as representative dot plots for validation of L1CAM expression in IGROV1 WT, ΔL1CAM and ΔL1CAM rescue cells. **(D)** Corresponding Western blot results for validation of L1CAM expression in IGROV1 WT, ΔL1CAM and ΔL1CAM rescue cells. GAPDH was used as the loading control.

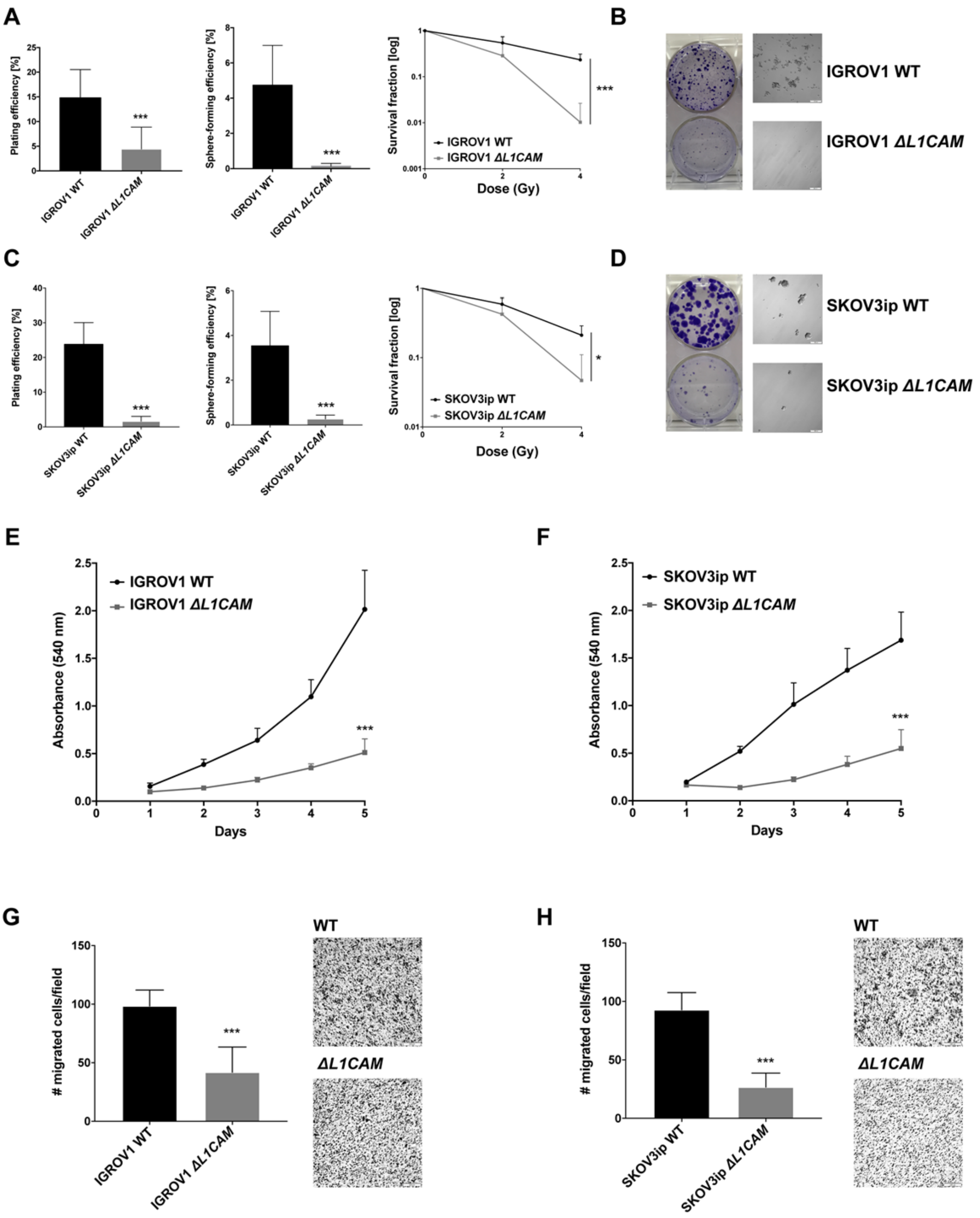

Figure S4. IGROV1 and SKOV3ip ΔL1CAM cells showed reduced clonogenic properties, spherogenic capacity, radioresistance, proliferation rate and migration ability and in comparison to wild type cells. **(A)** Clonogenic capacity (left graph), spherogenic capacity (middle graph) and radiation responsiveness (right graph) of IGROV1 wild type and ΔL1CAM cells. Each experiment has been performed three times in triplicates and data are expressed as means ± SD. Student t-test (two-tailed, unpaired); ***p < 0.001. **(B)** Representative pictures of 2D colonies and 3D spheres of IGROV1 wild type and ΔL1CAM cells. **(C)** Clonogenic capacity (left graph), spherogenic capacity (middle graph) and radiation responsiveness (right graph) of SKOV3ip wild type and ΔL1CAM cells. Each experiment has been performed three times in triplicates and data are expressed as means ± SD. Student t-test (two-tailed, unpaired); * p < 0.03 and ***p < 0.001. **(D)** Representative pictures of 2D colonies and 3D spheres of SKOV3ip wild type and ΔL1CAM cells. **(E and F)** After seeding, IGROV1 **(E)** and SKOV3ip **(F)** wild type and ΔL1CAM cells were treated with MTT for five consecutive days. Absorbance was measured at 540 nm. Error bars indicate SD. Two-way ANOVA; ***p < 0.001. **(G and H)** Representative photos and statistical plots of migration assays for IGROV1 **(G)** and SKOV3ip **(H)** wild type and ΔL1CAM cells. Following 24 h incubation, cells that migrated through the insert were counted in five random fields. Student t-test (two-tailed, unpaired); ***p < 0.001.

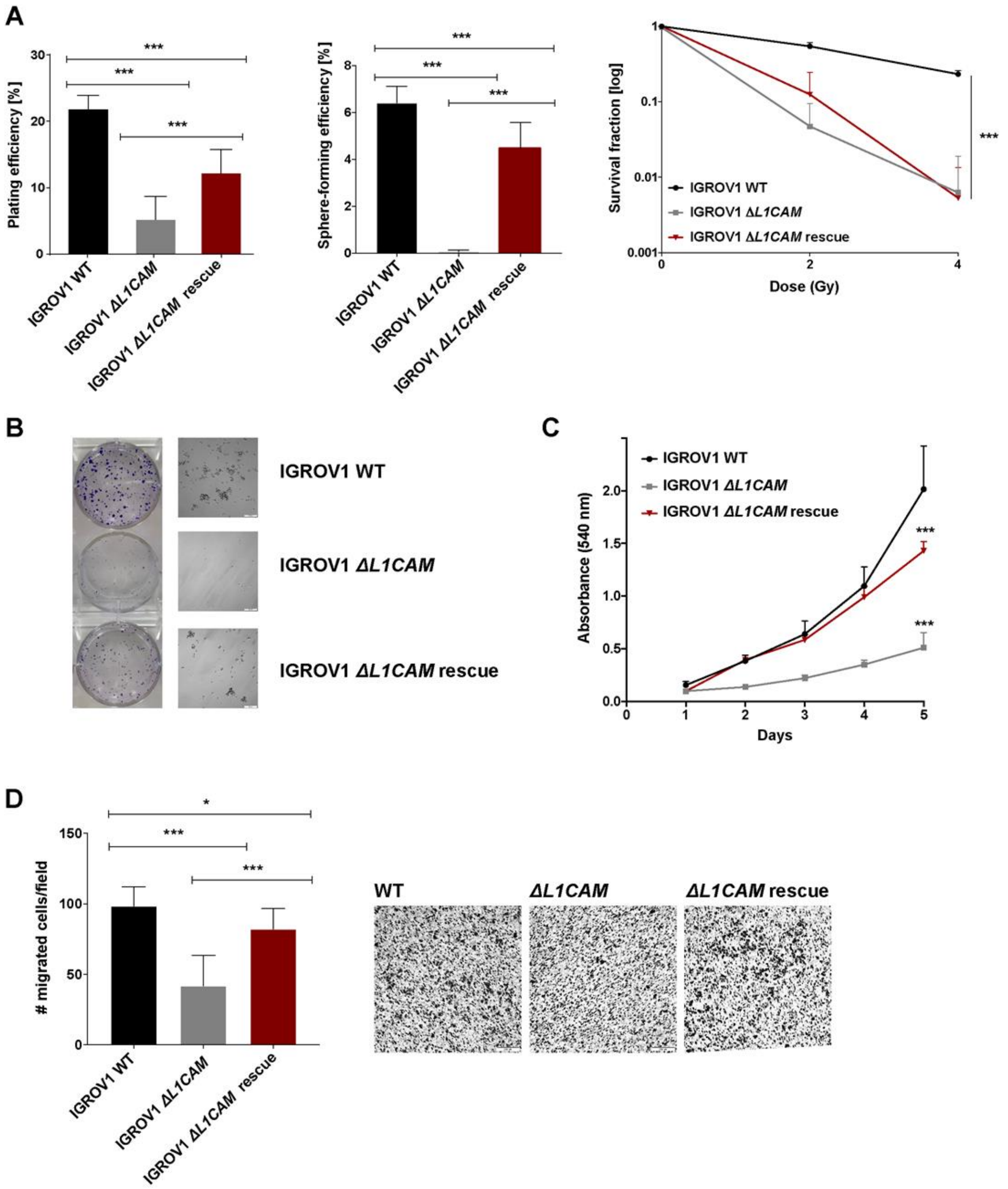

Figure S5. Constitutive L1CAM expression in IGROV1 ΔL1CAM cells partially restored clonogenic properties, spherogenic capacity, radioresistance, migration ability and proliferation as compared to wild type cells. **(A)** Clonogenic capacity (left graph), spherogenic capacity (middle graph) and radiation responsiveness (right graph) of IGROV1 wild type, ΔL1CAM and ΔL1CAM rescue cells. Each experiment has been performed three times in triplicates and data are expressed as means ± SD. One-way ANOVA; ***$p < 0.001$. **(B)** Representative pictures of 2D colonies and 3D spheres. **(C)** After seeding, for IGROV1 wild type, ΔL1CAM and ΔL1CAM rescue cells were treated with MTT for five consecutive days. Absorbance was measured at 540 nm. Error bars indicate SD. Two-way ANOVA; ***$p < 0.001$ **(D)** Statistical plots and representative photos and of migration assays for IGROV1 wild type, ΔL1CAM and ΔL1CAM rescue cells. Following 24 h incubation, cells that migrated through the insert were counted in five random fields. One-way ANOVA; * $p < 0.03$ and ***$p < 0.001$.

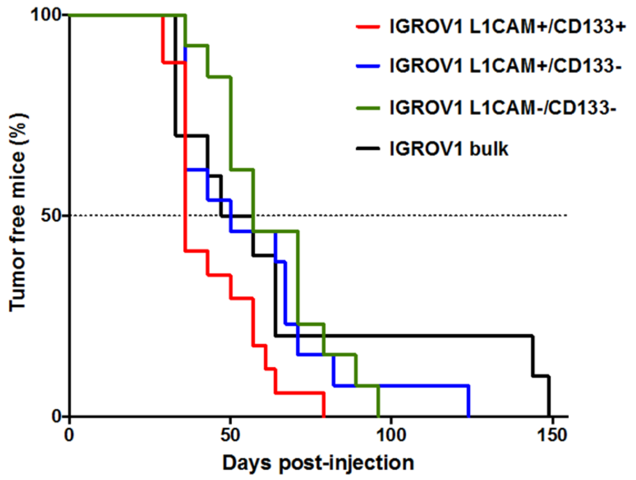

Figure S6. IGROV1 L1CAM+/CD133+ population showed shorter tumor latency *in vivo*. CD1 nude mice (n=3) were palpated weekly to detect subcutaneous tumor growth. The graph shows the number of days to tumor detection versus the percentage of tumor-free mice for each group. Tumors appeared with median latencies of 36 days (L1CAM+/CD133+), 50 days (L1CAM+/CD133-), 57 days (L1CAM-/CD133-) and 52 days (IGROV1 bulk).

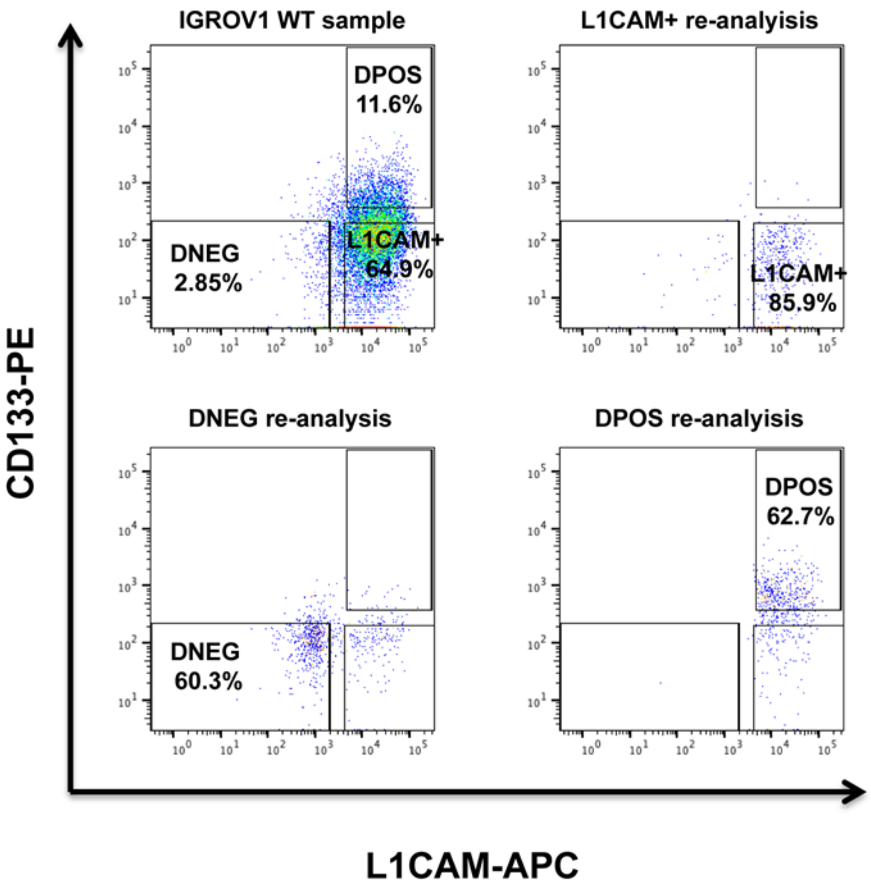

Figure S7. Re-analysis of IGROV1 cells isolated by FACS for in vivo limiting dilution assay (LDA). After FACS, 600 cells derived from the corresponding populations (L1CAM+: L1CAM+/CD133-; DNEG: L1CAM-/CD133-; DPOS: L1CAM+/CD133+) were re-analyzed by flow cytometry for L1CAM and CD133 purity before injection into mice. Gating was performed as exemplified, accordingly to isotype-matched IgG controls.

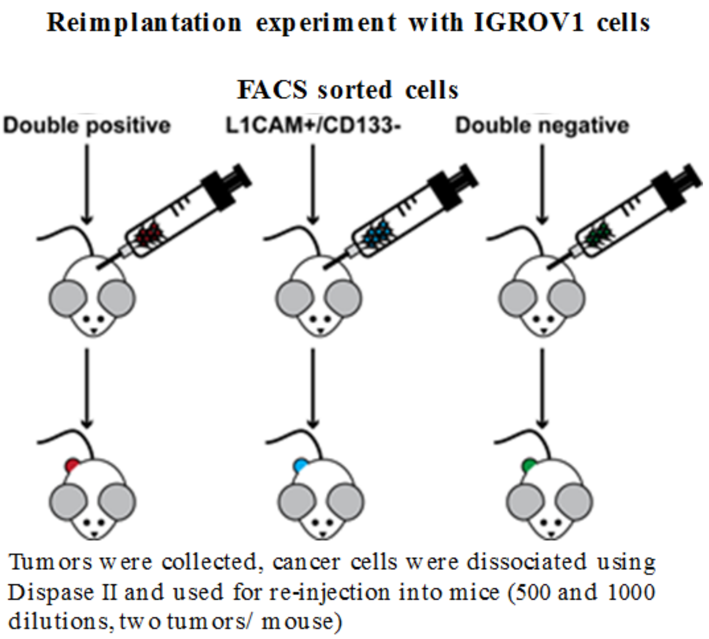

Figure S8. Scheme of the re-implantation experiment.

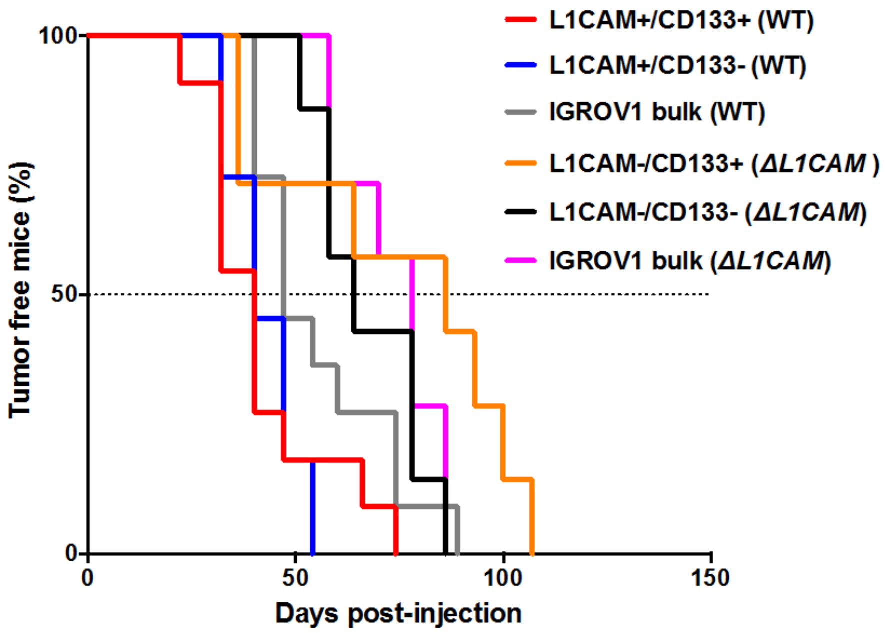

Figure S9. IGROV1 L1CAM-/CD133- cells showed significantly longer tumor latency *in vivo* compared to L1CAM+/CD133+ population. CD1 nude mice (n=6) were palpated weekly to detect subcutaneous tumor growth. The graph shows the number of days to tumor detection versus the percentage of tumor-free mice for each group. Tumors appeared with median latencies of 40 days (L1CAM+/CD133+ and L1CAM+/CD133-), 47 days (IGROV1 WT), 86 days (L1CAM-/CD133+), 64 days (L1CAM-/CD133-) and 78 days (IGROV1 ΔL1CAM).

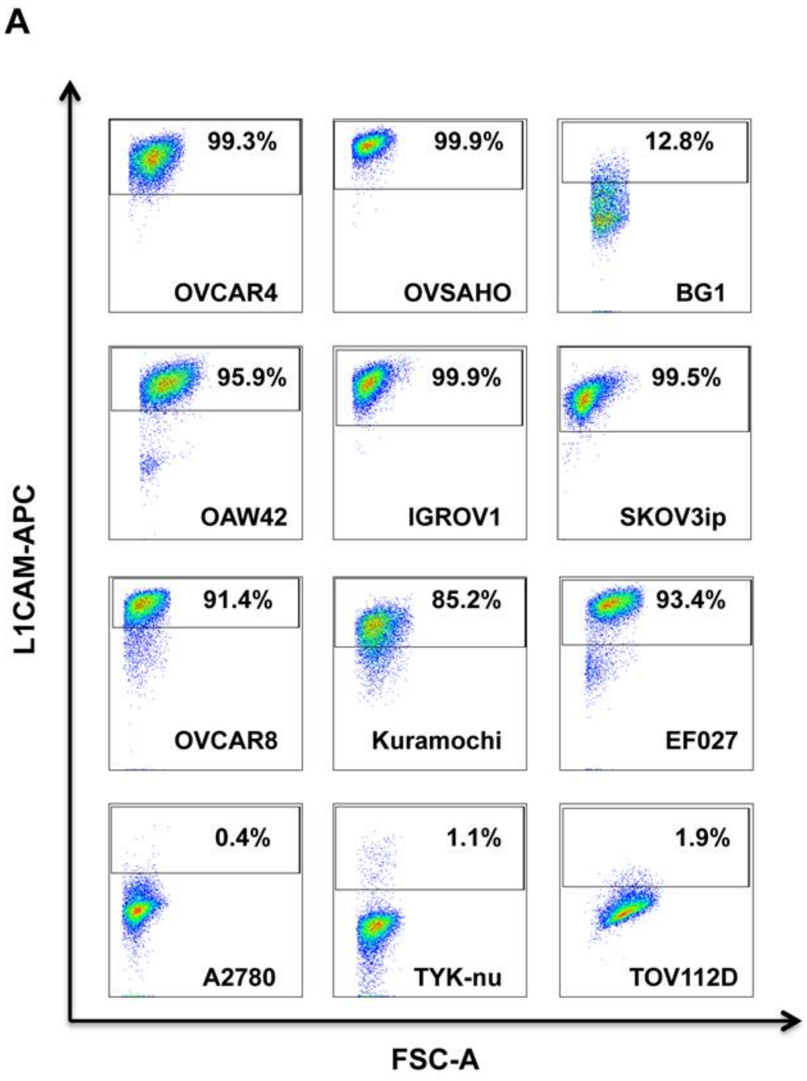

Figure S10. L1CAM expression in a panel of ovarian cancer cell lines classified based on EMT phenotype. **(A)** Representative flow cytometry for L1CAM expression in 12 cells lines shown as pseudocolor dot plots. Gating was performed as exemplified, accordingly to isotype-matched IgG controls. The numbers indicate the percentage of antigen-expressing cells in the sample population.

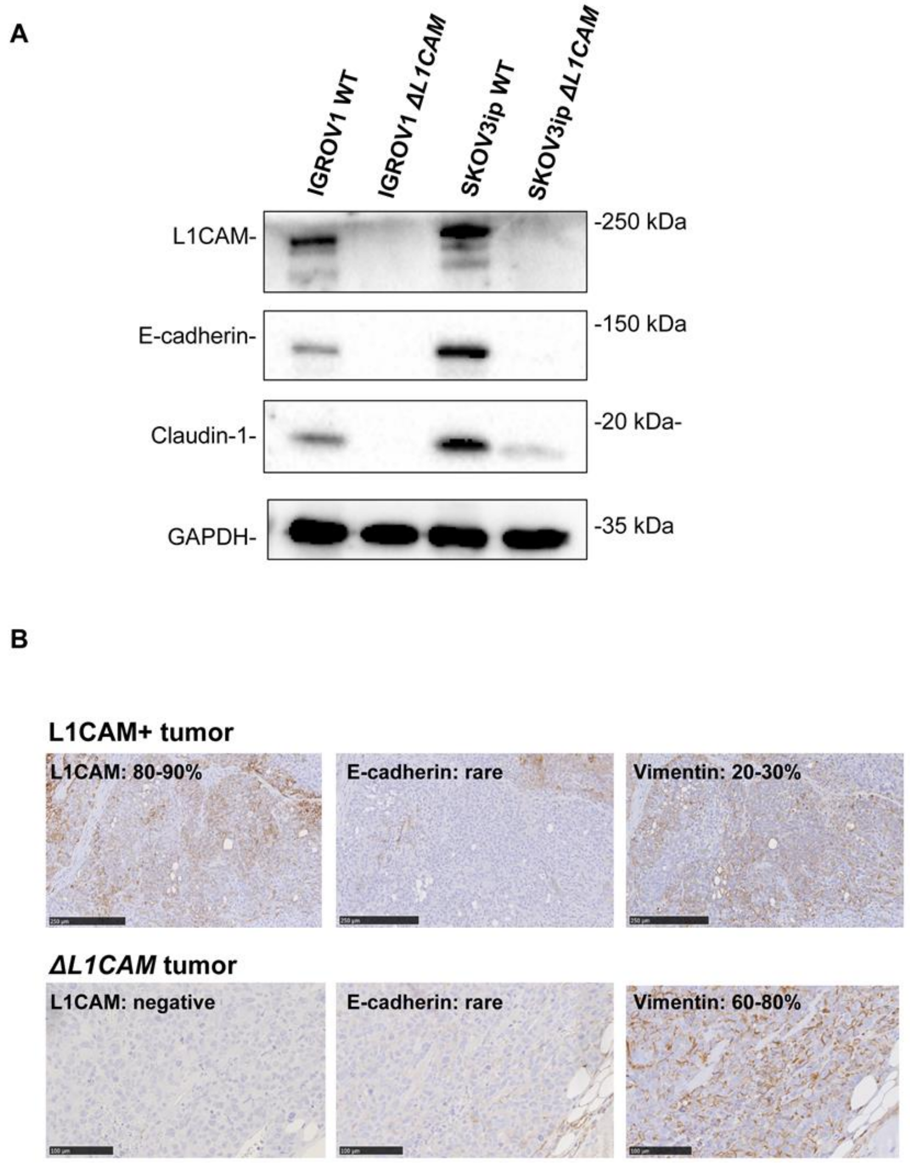

Figure S11. The loss of L1CAM shifted the cells towards a more mesenchymal-like state characterized by loss of E-cadherin and claudin-1. **(A)** Western blot data showing loss of E-cadherin and claudin-1 expression in ΔL1CAM IGROV1 and SKOV3ip cells. GAPDH was used as the loading control. **(B)** Immunohistochemistry of IGROV1 WT and IGROV1 ΔL1CAM tumors for L1CAM, E-cadherin and vimentin. Magnification x10 (IGROV1 WT) and x20 (IGROV1 ΔL1CAM).

A

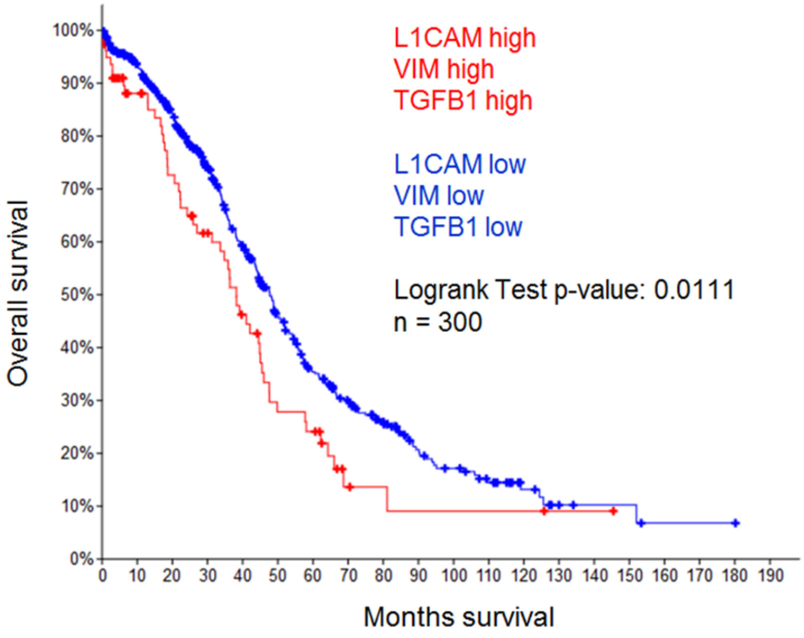

B

**Gene A**	**Gene B**	**p-Value**	**q-Value**	**Tendency**
**VIM: EXP>1**	**TGFB1: EXP>1**	**0.018**	**0.054**	**Co-occurrence**
**L1CAM: EXP>1**	**TGFB1: EXP>1**	**0.038**	**0.056**	**Co-occurrence**
**L1CAM: EXP>1**	**VIM: EXP>1**	**0.584**	**0.584**	**Co-occurrence**

Figure S12. Analysis of the TCGA (PanCancer Atlas, RNAseq expression) dataset. **(A)** Low expression of gene signature including L1CAM, vimentin and TGF-β1 genes is significantly associated with better overall survival of ovarian cancer patients. **(B)** L1CAM, vimentin and TGF-β1 expression has a strong tendency toward co-occurrence in tumor tissues.

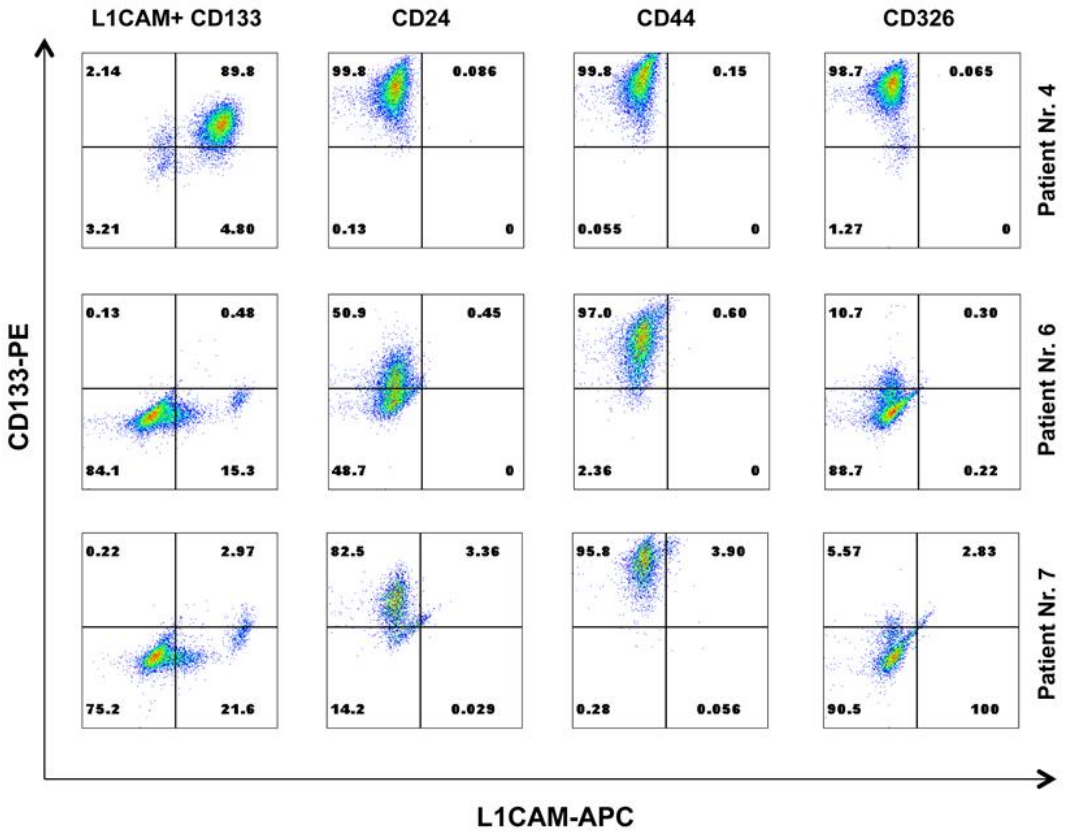

Figure S13. Expression of putative cancer stem cell markers in human ovarian cancer ascites samples is heterogeneous. Representative flow cytometry expression for putative CSC marker of three ascites samples (patient Nr. 4, patient Nr. 6 and patient Nr.7) shown as pseudocolor dot plots. Gating was performed as exemplified, accordingly to isotype-matched IgG controls. The numbers indicate the percentage of antigen-expressing cells in the sample population.

SUPPLEMENTARY TABLES

Table S1

Table providing details of oligonucleotides used in this study.
Name of oligonucleotide, DNA sequence and applied method for specific oligonucleotide are provided.

**Oligonucoleotide name**	**Sequences (5′—3′)**	**Method**
sgRNA1_Forward	caccgGAGGGGTTGCACTCATGAGT	CRISPR-*Cas9*
sgRNA1_Reverse	aaacACTCATGAGTGCAACCCCTCC	CRISPR-*Cas9*
sgRNA2_Forward	caccgTGTGGGGGGTGTTACCGTGA	CRISPR-*Cas9*
sgRNA2_Reverse	aaacTCACGGTAACACCCCCCACAC	CRISPR-*Cas9*
Human U6_Forward	GAGGGCCTATTTCCCATGATTCC	Cloning
PCR_1,2_Forward (CR) *ΔL1CAM*	CACCTCAGCCTCCCAAACTA	Genotyping
PCR_1,3_Reverse (CR) *ΔL1CAM*	TGGTGTGTGTTTCCACCTGT	Genotyping
PCR_2,3_Reverse (WT) *ΔL1CAM*	TACCCAACGTCCTGGCTATC	Genotyping
T7_Forward	TAATACGACTCACTATAGGG	DNA Sequencing
EGFP_Forward	CAACGAGAAGCGCGATC	DNA Sequencing
L1CAM-HA__XbaI _Forward	GAATCTAGAATGGTCGTGGCGCTGCG	Cloning
L1CAM-HA_ NhEI _Reverse	CAAGCTAGCTTCTAGGGCCACGGCAG	Cloning
L1CAM_qPCR_Forward	CAGCCCGAGCGGTGG	RT-qPCR
L1CAM_qPCR_Reverse	ATCTGGATAAGCAGGCAGGG	RT-qPCR
LIN28_Forward	GAAGCGCAGATCAAAAGGAG	RT-qPCR
LIN28_Reverse	GCTGATGCTCTGGCAGAAGT	RT-qPCR
OCT4_Forward	CTTCGCAAGCCCTCATTTC	RT-qPCR
OCT4_Reverse	GAGAAGGCGAAATCCGAAG	RT-qPCR
ABCG2_Forward	CCAAAGACATTGATAAAGCCATAA	RT-qPCR
ABCG2_Reverse	CACGCCATAGCAATTCACC	RT-qPCR

CXCR4_Forward	GCCTTATCCTGCCTGGTATTGTC	RT-qPCR
CXCR4_Reverse	GCGAAGAAAGCCAGGATGAGGAT	RT-qPCR
Vimentin_Forward	ACACCCTGCAATCTTTCAGACA	RT-qPCR
Vimentin _Reverse	GATTCCACTTTGCGTTCAAGGT	RT-qPCR
β-catenin_Forward	AAAATGGCAGTGCGTTTAG	RT-qPCR
β-catenin_Reverse	TTTGAAGGCAGTCTGTCGTA	RT-qPCR
TGF-β1_Forward	GCATTAGCTTGAAGCACTACAGGA	RT-qPCR
TGF-β1_Reverse	GCACAAGGCTCACATCTCATTATG	RT-qPCR
NBS1_Forward	AGACCAACTCCATCAGAAACTAC	RT-qPCR
NBS1_Reverse	AATGAGGGTGTAGCAGGT TG	RT-qPCR
RAD50_Forward	CGAAGTACCTATCGTGGACAAG	RT-qPCR
RAD50_Reverse	GATCGTCCTCGCATATCCAAG	RT-qPCR
GAPDH_Forward	AGCCACATCGCTCAGACAC	RT-qPCR
GAPDH_Reverse	GCCCAATACGACCAAATCC	RT-qPCR

Table S2

Expression of putative cancer stem cell markers of IGROV1 tumor cells before re-injection into nude mice.

For *in vivo* serial passaging experiment, tumors were collected from CD1 nude mice previously inoculated with sorted IGROV1 cells and single-cell suspensions were obtained using Dispase II. The expression of different cell surface markers was analyzed by flow cytometry before re-injection into mice. The numbers indicate the percentage of antigen-expressing cells in the sample population ± SD of different experiment.

	% L1CAM+	% CD133+	% L1CAM+CD133+	% CD24+	% CD44+	% CD326+
**IGROV1 DNEG (n=3)**	9.1 ± 1.1	1.5 ± 1.1	0.9 ± 0.8	91.4 ± 6.6	59.2 ± 16.9	43.7 ± 40.6
**IGROV1 DPOS (n=2)**	61.7 ± 1.6	14.6 ± 4.9	13.3 ± 2.1	95.2 ± 5.3	98.4 ± 1.8	4.7 ± 0.9
**IGROV1 L1CAM+ (n=2)**	69.7 ± 12.4	2.0 ± 2.6	2.1 ± 1.5	98.7 ± 0.2	97.6 ± 0.6	13.8 ± 6.4
**IGROV1 CONTROL (n=2)**	65.1 ± 1.6	0.6 ± 0.4	0.8 ± 0.6	96.8 ± 2.8	91.2 ± 9.9	11.4± 8.9

**Color key [% of positive cells]**	0-5%	5-25%	25-50%	50-75%	>75%

Table S3

Tumor-initiating capacity of L1CAM/CD133 cell populations isolated from IGROV1 wild type and *ΔL1CAM* cell lines.

Tumor initiation assay in CD1 nude mice (n = 6; 2 tumors per mouse) was performed with wild type and *ΔL1CAM* ovarian cancer IGROV1 cells isolated by FACS based on L1CAM and CD133 expression. Five hundred cells were subcutaneously injected into CD1 nude mice. Tumor take was determined as number of mice with palpable tumors at day 153.

	Tumor take	Tumor latency (days)
**L1CAM+/CD133+ (WT)**	11/12	22-74
**L1CAM+/CD133- (WT)**	12/12	32-74
**IGROV1 bulk (WT)**	12/12	40-89
**L1CAM-/CD133+ (*ΔL1CAM*)**	7/12	36-107
**L1CAM-/CD133- (*ΔL1CAM*)**	7/12	51-86
**IGROV1 bulk (*ΔL1CAM*)**	4/12	58-86

Table S4

Expression of putative cancer stem cell markers in ovarian cancer cell lines.

The expression of different markers was analyzed by flow cytometry. The numbers indicate the percentage of antigen-expressing cells in the sample population ± SD of three independent experiments.

Cell lines	% L1CAM+	% CD133+	% L1CAM+CD133+	% CD24+	% CD44+	% CD326+	% ALDH+
OVCAR4	98.2 ± 1.8	95.1 ± 3.5	93.5 ± 2	98.3 ± 2.8	98 ± 1.7	99.6 ± 0.6	0.2 ± 0.3
OVCAR5	19.8 ± 4.2	5.2 ± 1.2	0.6 ± 0.5	93.2 ± 11	98 ± 3.4	94.6 ± 9.2	39.4 ± 22
CAOV3	99.3 ± 1	8.9 ± 2.4	8.6 ± 2.4	100 ± 0.05	100	100	0.7 ± 0.6
OVSAHO	98.6 ± 1.2	1.4 ± 0.6	1.4 ± 0.6	99.5 ± 0.05	31.9 ± 1.8	99.9 ± 0.05	8.9 ± 7.8
BG1	9.1 ± 5.3	3.5 ± 1.3	1.5 ± 1.4	92.2 ± 6.2	97.4 ± 3.6	99.9 ± 0.1	8.4 ± 5.6
OAW42	98.9 ± 1.1	98.7 ± 0.8	98.1 ± 0.9	99 ± 0.7	98.2 ± 1.8	98.8 ± 0.8	0.06 ± 0.042
IGROV1	99.9 ± 0.1	9.8 ± 0.4	9.8 ± 0.4	99.9 ± 0.1	99.9 ± 0.05	60.9 ± 1.9	29.5 ± 0.47
SKOV3ip	88.2 ± 0.1	1.3 ± 0.5	0.07 ± 0.04	98.4 ± 1.3	98.7 ± 1.7	95.9 ± 4.9	4.22 ± 2.0
OVCAR8	91.4 ± 8.5	2.4 ± 2.6	2.1 ± 2.6	97.8 ± 3.7	97.7 ± 3.9	94.6 ± 9.2	0.7 ± 0.5
Kuramochi	81 ± 5.4	1 ± 0.3	0.8 ± 0.3	99.6 ± 0.4	88.1 ± 5.9	61.7 ± 2.3	3 ± 2.9
EFO27	98.6 ± 1.4	1.2 ± 0.6	1.2 ± 0.6	96.2 ± 6.3	100	92.9 ± 1.9	5.5 ± 4.1
A2780	0.3 ± 0.1	0.5 ± 0.4	0.01 ± 0.01	50.9 ± 8.5	0.3 ± 0.2	4.9 ± 4.7	19.1 ± 1.4
TYK-nu	0.8 ± 0.3	0.7 ± 0.5	0.3 ± 0.2	6.1 ± 2.2	94.8 2.8	0.15 ± 0.02	0.9 ± 0.9
TOV112D	2.3 ± 0.8	1.2 ± 0.2	0.4 ± 0.6	23.6 ± 4.6	0.5 ± 0.3	3.3 ± 1.7	5.2 ± 0.3

Color key [% of positive cells]	0-5%	5-25%	25-50%	50-75%	>75%

Table S5

Human cancer cells derived from ovarian cancer patients' ascites and ovarian cancer cell lines show heterogeneous expression of different cell surface markers.

The expression of putative CSC markers was analyzed by flow cytometry. FIGO stage for each ovarian cancer patients' ascites is provided. The numbers indicate the percentage of antigen expressing-cells in the sample population ± SD of three independent experiments. Due to the limited number of cells, the expression of markers in patient-derived samples was measured only once.

Patient Nr.	L1CAM [%]	CD133 [%]	L1CAM+CD133 [%]	CD24 [%]	CD44 [%]	CD326 [%]	ALDH [%]	FIGO stage
1	9.2	0.4	0.2	1.3	99.7	0.6	31.8	IV
2	21.5	0.6	0.5	34.1	99.8	7.6	43.4	III
3	2.1	0.2	0.1	7	99.8	0.3	42.2	III
4	94.9	97	93.6	99.9	100	99.9	0	III
5	31.1	1.2	0.8	98.7	99.9	10.43	43.6	III
6	15.9	0.6	0.5	51	97	10.8	0	III
7	34.3	1.8	1.5	81	96.5	5.2	3.4	III
8	16.7	1.4	1.3	58.5	99.4	2.4	28.8	IV
9	27.6	0.7	0.7	91.8	99.8	18.1	21.9	IV
10	5.3	0.4	0.1	10.7	99.3	1.1	0	III
11	25.4	1.1	1	16.7	99.2	0.1	0	III
12	9.7	0	0	0.7	93.9	0.1	30.1	IV

Table S6

Clinicopathological data of ovarian cancer patients.

Clinicopathological data of patients include age, type of therapy received, diagnose, tumor histotype, grade and FIGO stage classification.

Patient Nr.	Age	Primary/Neo-adjuvant	Diagnose	Histotype	Grade	FIGO stage
1	67	Neo-adjuvant	Ovarian	Serous	3	IV
2	76	Primary	Ovarian	Serous	3	III
3	46	Primary	Ovarian	Serous	3	III
4	54	Primary	Ovarian	Mixed	3	III
5	69	Primary	Ovarian	Clear cell	3	III
6	71	Primary	Ovarian	Serous	3	III
7	78	Neo-adjuvant	Tubal	Serous	3	III
8	45	Primary	Tubal	Serous	3	IV
9	70	Primary	Ovarian	Serous	3	IV
10	48	Primary	Tubal	Serous	3	III
11	58	Primary	Tubal	Serous	3	III
12	78	Primary	Ovarian	Serous	3	IV

References

1. Grünberg, J.; Knogler, K.; Waibel, R.; Novak-Hofer, I. High-yield production of recombinant antibody fragments in HEK-293 cells using sodium butyrate. *Biotechniques* **2003**, 34, 968–972.
2. Ran, F.A.; Hsu, P.D.; Wright, J.; Agarwala, V.; Scott, D.A.; Zhang, F. Genome engineering using the CRISPR-Cas9 system. *Nat. Protoc*. **2013**, 8, 2281–2308.

© 2020 by the authors. Licensee MDPI, Basel, Switzerland. This article is an open access article distributed under the terms and conditions of the Creative Commons Attribution (CC BY) license (http://creativecommons.org/licenses/by/4.0/).
